# *Insm1* promotes endocrine cell differentiation by modulating the expression of a network of genes that includes *Neurog3* and *Ripply3*

**DOI:** 10.1242/dev.104810

**Published:** 2014-08

**Authors:** Anna B. Osipovich, Qiaoming Long, Elisabetta Manduchi, Rama Gangula, Susan B. Hipkens, Judsen Schneider, Tadashi Okubo, Christian J. Stoeckert, Shinji Takada, Mark A. Magnuson

**Affiliations:** 1 Center for Stem Cell Biology, Vanderbilt University School of Medicine, Nashville, TN 37232, USA; 2 Department of Molecular Physiology and Biophysics, Vanderbilt University School of Medicine, Nashville, TN 37232, USA; 3 Department of Animal Science, Cornell University, Ithaca, NY 14850, USA; 4 Penn Center for Bioinformatics, Department of Genetics, University of Pennsylvania School of Medicine, Philadelphia, PA 19104, USA; 5 Department of Laboratory Animal Science, Kitasato University School of Medicine, Sagamihara, 252-0374, Japan; 6 Okazaki Institute for Integrative Bioscience, National Institutes of Natural Sciences, Okazaki, Aichi, 444-8787, Japan

**Keywords:** Pancreas development, Endocrine progenitor cells, Gene expression, Transcription factors, Mouse

## Abstract

Insulinoma associated 1 (*Insm1*) plays an important role in regulating the development of cells in the central and peripheral nervous systems, olfactory epithelium and endocrine pancreas. To better define the role of *Insm1* in pancreatic endocrine cell development we generated mice with an *Insm1^GFPCre^* reporter allele and used them to study *Insm1*-expressing and null populations. Endocrine progenitor cells lacking *Insm1* were less differentiated and exhibited broad defects in hormone production, cell proliferation and cell migration. Embryos lacking *Insm1* contained greater amounts of a non-coding *Neurog3* mRNA splice variant and had fewer *Neurog3*/*Insm1* co-expressing progenitor cells, suggesting that *Insm1* positively regulates *Neurog3*. Moreover, endocrine progenitor cells that express either high or low levels of *Pdx1*, and thus may be biased towards the formation of specific cell lineages, exhibited cell type-specific differences in the genes regulated by *Insm1*. Analysis of the function of *Ripply3*, an *Insm1*-regulated gene enriched in the *Pdx1*-high cell population, revealed that it negatively regulates the proliferation of early endocrine cells. Taken together, these findings indicate that in developing pancreatic endocrine cells *Insm1* promotes the transition from a ductal progenitor to a committed endocrine cell by repressing a progenitor cell program and activating genes essential for RNA splicing, cell migration, controlled cellular proliferation, vasculogenesis, extracellular matrix and hormone secretion.

## INTRODUCTION

The genetic program responsible for the generation of pancreatic endocrine cells from endocrine progenitors remains incompletely understood (Oliver-Krasinski and Stoffers, 2008; Rieck et al., 2012; Stanger and Hebrok, 2013). Beginning at around embryonic day (E) 9-9.5 in the mouse, and peaking around E15.5, pancreatic epithelial cells express *Neurog3*, which encodes a master regulatory factor that activates a cascade of secondary transcription factor genes, including *Insm1*, *Neurod1*, *Pax4*, *Arx* and *Rfx6*. Together, these and other transcription factors orchestrate the formation of the five endocrine cell types found in adult islets (Huang et al., 2000; Schwitzgebel et al., 2000; Gu et al., 2002; Collombat et al., 2003; Smith et al., 2003; Mellitzer et al., 2006; Soyer et al., 2010). Neurog3 triggers pre-endocrine cells to delaminate from a pancreatic ductal epithelium by an epithelial-to-mesenchymal transition (EMT), lose basal-apical cell polarity and epithelial adhesions, then migrate and aggregate to form islets of Langerhans (Cole et al., 2009; Gouzi et al., 2011). Moreover, *Neurog3* expression coincides both with a markedly lower rate of cell proliferation and an increase in the expression of cyclin-dependent kinase inhibitors such as Cdkn1c (p57^Kip2^) and Cdkn1a (p21^Cip1^) (Georgia et al., 2006; Miyatsuka et al., 2011).

Multiple transcription factors regulate pancreatic endocrine cell development, and they have interacting and sometimes opposing functions. For instance, Arx drives the formation of glucagon-producing α-cells. In its absence, there is a preponderance of insulin-producing β-cells and somatostatin-producing δ-cells. Similarly, Pax4 opposes the effect of Arx and is essential for the formation of β-cells, since mice lacking this factor are characterized by an expansion in α-cells (Sosa-Pineda et al., 1997; Collombat et al., 2003). Moreover, nascent β-cells express higher amounts of Pdx1, a transcription factor crucial for the early specification of pancreatic epithelium, compared with other pre-endocrine cells (Ohlsson et al., 1993; Ahlgren et al., 1998; Fujitani et al., 2006; Nishimura et al., 2006; Gannon et al., 2008). Other transcription factors important for β-cell specification and development, such as Nkx2.2, Neurod1, Nkx6.1, Mafb and Mafa, also function in an interrelated manner (Sosa-Pineda et al., 1997; Sussel et al., 1998; Nishimura et al., 2006; Nelson et al., 2007; Schaffer et al., 2013).

The expression of *Insm1*, which encodes a zinc-finger transcription factor, is activated by the binding of Neurog3 to sites within its promoter (Mellitzer et al., 2006). Endocrine cell differentiation is markedly perturbed in mice lacking *Insm1* (Gierl et al., 2006)*.* In the absence of this factor, there is a reduction in the number of insulin-expressing cells, with many cells lacking any hormone expression*.* In addition to being expressed in developing endocrine cells throughout the gut, *Insm1* is also expressed in the developing central nervous system, where it contributes to the formation and expansion of intermediate (basal) neural progenitors from early apical progenitor cells (Farkas et al., 2008), in the peripheral neural system and in the olfactory epithelium, where it is involved in regulating the differentiation of neurogenic progenitor cells (Wildner et al., 2008; Rosenbaum et al., 2011).

The acquisition of robust quantitative global gene transcription datasets, which are necessary for understanding the gene regulatory network that dictates the formation and function of endocrine cells, requires the combined use of fluorescent reporter alleles, fluorescence-activated cell sorting (FACS) and next-generation sequencing technology. To this end, we have derived mice containing an *Insm1^GFPCre^* reporter allele that enabled us to isolate highly purified populations of *Insm1*-expressing and -deficient pre-endocrine cells, and to characterize these cell populations by RNA-Seq. In addition, we performed multi-channel FACS to isolate and differentially characterize *Insm1-*positive cell populations that express either high or low levels of *Pdx1*. In doing so, we identified many genes that are likely to contribute to both the formation and function of pancreatic endocrine cell types. In addition, the function of *Ripply3* and the alternative RNA processing of *Neurog3* mRNA were examined. Together, these studies provide multiple new insights into the gene regulatory network controlling pancreatic endocrine cell formation and function.

## RESULTS

### Generation of *Insm1^GFPCre^* reporter mice

**Fig. 1.**
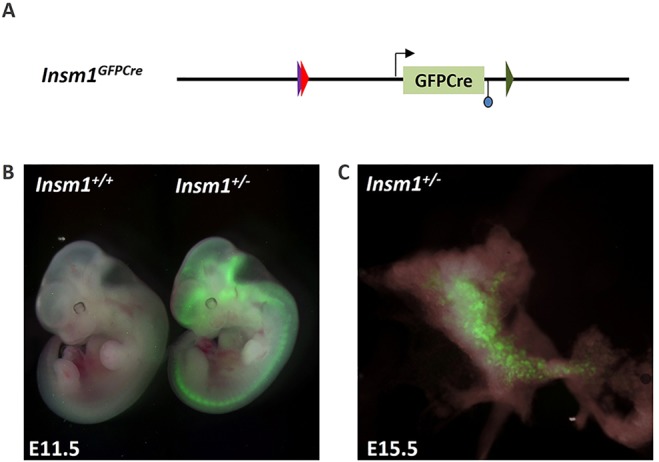
**The *Insm1^GFPCre^* allele.** (A) Schematic of the *Insm1^GFPCre^* allele. *Insm1* coding sequences were replaced with those encoding GFPCre using combined gene targeting/recombinase-mediated cassette exchange (RMCE) as described in supplementary material Fig. S1. The triangles represent heterotypic loxP sites and the circle a remnant FLP recognition target (FRT) site. (B) Green fluorescence in a whole mouse embryo at E11.5 broadly marks the neural system. (C) Green fluorescence in a pancreas at E15.5 marks pre-endocrine cells. Fluorescence images were overlaid with images taken with white light.

A two-step strategy utilizing both gene targeting and recombinase-mediated cassette exchange (RMCE) was used to derive mice that express a green fluorescent protein-Cre fusion protein (*GFPCre*) under control of the endogenous *Insm1* gene locus (Fig. 1A; supplementary material Fig. S1A-F). Insertion of *GFPCre* sequences into the *Insm1* gene locus disrupted Insm1 protein expression, as confirmed by western blot analysis of homozygous null embryos (supplementary material Fig. S1F). Mice heterozygous for this allele (hereafter termed *Insm1^+/−^*) appeared normal, whereas animals that were homozygous for *Insm1^GFPCre^* (hereafter termed *Insm1^−/−^*) died between E15.5 and E18.5 due to defects in catecholamine biosynthesis and secretion (Wildner et al., 2008). Green fluorescence was observed in pancreatic pre-endocrine cells (Fig. 1C) and GFPCre was readily detectable by immunofluorescence in islet cells after birth [at postnatal day (P) 1, P7 and P60; data not shown] and in the developing central nervous system between E9.5 and E18.5 (Fig. 1B). *GFPCre* expression was also detected in the peripheral nervous system and gut endocrine cells (data not shown). Co-staining with anti-GFP and anti-Insm1 antibodies at E15.5-18.5 in *Insm1^+/−^* pancreata showed that the majority of *Insm1*-positive cells co-expressed both proteins (supplementary material Fig. S2). Functionality of the Cre portion of the GFPCre fusion was also confirmed (data not shown).

### *Insm1* knockout mice have altered pancreatic hormone cell differentiation, replication, size and migration

To investigate the role of *Insm1* in pancreas development we quantified the percentage of different pancreatic hormone-positive cells among *GFPCre*-expressing endocrine cells in *Insm1* heterozygous and knockout animals at E18.5 (supplementary material Fig. S3). Consistent with the results of Gierl et al. (2006), 54% of endocrine cells expressed insulin in heterozygous animals, whereas only 8% of *GFPCre*-expressing cells were insulin positive (a 6-fold decrease) in *Insm1^−/−^* embryos. There were also less pronounced but significant decreases in cells expressing glucagon (from 24% to 11%), somatostatin (from 11% to 7%) and ghrelin (from 8% to 5%) in the null embryos. Also, the number of pancreatic polypeptide-positive cells increased from 7% to 12% in the knockout animals, as is also consistent with the findings of Gierl et al. (2006).

**Fig. 2.**
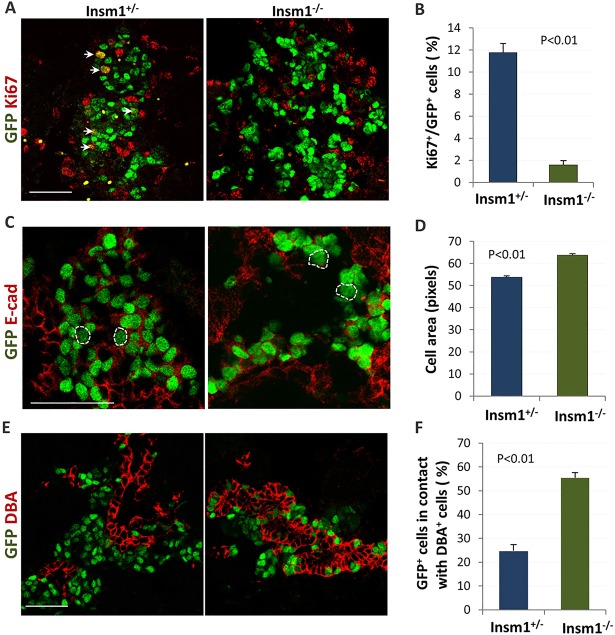
**Impaired proliferation, cell size and migration of pre-endocrine cells in *Insm1* knockout mice.** (A) Immunofluorescence labeling of pancreatic tissues from *Insm1^+/−^* and *Insm1^−/−^* mice at E18.5 with anti-GFP antibodies (green) and antibodies against cell proliferation marker Ki67 (red). Arrows indicate cells co-expressing GFP and Ki67. (B) Percentage of Ki67-positive cells among GFP-positive cells demonstrates a proliferation defect in *Insm1^−/−^* pancreas. (C) Immunofluorescence labeling of pancreatic tissues from *Insm1^+/−^* and *Insm1^−/−^* mice at E18.5 with anti-GFP antibodies (green) and antibodies against cell surface marker E-cadherin (red) enabled the measurement of endocrine cell area. Representative cells for each phenotype are outlined. (D) Quantification of cell area shows increased cell size in *Insm1^−/−^* endocrine cells. (E) Immunofluorescence labeling of pancreatic tissues from *Insm1^+/−^* and *Insm1^−/−^* mice at E18.5 with anti-GFP antibodies (green) and the ductal marker DBA (red). (F) Quantification of the percentage of GFP-positive cells in contact with DBA-positive cells shows a defect in the migration of *Insm1^−/−^* endocrine cells away from the ducts. Error bars indicate s.e.m. (*n*≥6); *P*-values were determined by Student's *t*-test. Scale bars: 50 μm.

Since it has been suggested that Insm1 inhibits the progression of endocrine cells through the cell cycle (Zhang et al., 2009), we quantified the proliferation rates of *GFPCre*-positive endocrine cells by immunostaining for Ki67, a marker of cell proliferation (Fig. 2A). At E18.5 we observed up to a 7-fold decrease (from 11.6% to 1.5%) in the number of Ki67/GFPCre double-positive cells in heterozygous versus knockout pancreata (Fig. 2B). Concurrently, the size of the *Insm1^−/−^* endocrine progenitor cells is increased (Fig. 2C,D) and their shape becomes irregular. No differences in the number of apoptotic cells were observed between heterozygous and homozygous null animals by TUNEL assay (data not shown). To assess whether the proliferation defect is visible at an earlier stage, we also quantified proliferation rates at E15.5 (supplementary material Fig. S4). Although the majority of endocrine cells are postmitotic at E15.5, we were still able to detect a slight decrease in proliferation from 1.4% in *Insm1^+/−^* cells to 0.9% in *Insm1^−/−^* cells.

In addition to a reduced proliferation rate of *Insm1^−/−^* cells, we also observed that the GFPCre-positive endocrine cells remained closely associated with pancreatic ductal cells. We quantified the apparent cell migration defect by calculating the percentage of GFPCre-expressing cells that were in contact with ductal cells (DBA-positive cells) and found an increase (from 24% to 55%) in the knockout animals compared with heterozygous animals (Fig. 2E,F). This finding suggests that *Insm1*, in addition to its other roles, is necessary for the migration of the differentiating pre-endocrine cell away from pancreatic ductal regions.

### Identification of Insm1-dependent genes in developing endocrine cells

**Fig. 3.**
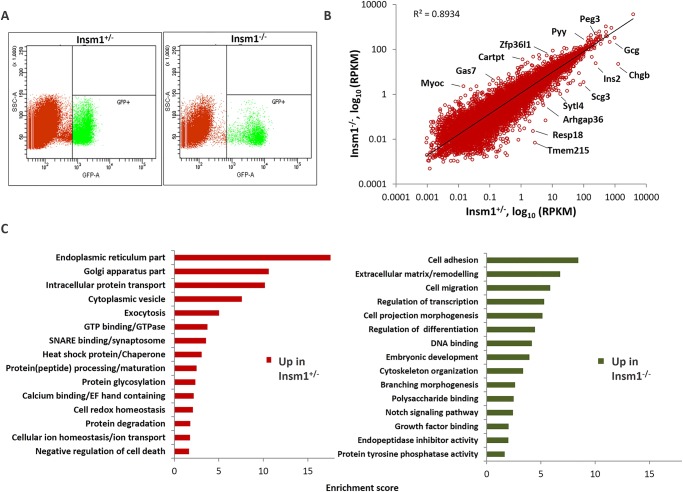
**RNA-Seq analysis of *Insm1^+/−^* and *Insm1^−/−^* endocrine progenitor cell populations at E15.5.** (A) Representative FACS profiles of *GFPCre*-positive *Insm1^+/−^* and *Insm1^−/−^* endocrine cell populations isolated at E15.5 and used for RNA isolation and RNA-Seq. (B) Analysis of RNA-Seq results demonstrates differential gene expression between *Insm1^+/−^* and *Insm1^−/−^* cells. Correlation plot of RPKM (reads per kilobase per million mapped reads, log_10_) values for identified genes in *Insm1^+/−^* and *Insm1^−/−^* cell populations shows that although the majority of reads fall along the line representing equal expression, a number of transcripts are upregulated in *Insm1^+/−^* or *Insm1^−/−^* cells. (C) Functional groupings of genes upregulated in *Insm1^+/−^* and *Insm1^−/−^* cell populations. Enrichment scores for functional gene ontology groups represent the geometric mean (in −log scale) of *P*-values in the corresponding annotation cluster.

To quantitatively assess the gene expression changes brought about by the absence of *Insm1*, we performed RNA-Seq using FACS-purified progenitor cells isolated at E15.5. On average, we obtained 13,456 (±2886) cells per *Insm1^+/−^* embryo and 8505 (±1602) cells per *Insm1^−/−^* embryo at E15.5, consistent with a proliferation defect. RNA-Seq resulted in the detection of 13,865 individual mRNAs with an RPKM >0.1. Pairwise comparison of the transcriptional profiles from *Insm1^+/−^* and *Insm1^−/−^* mice revealed that the expression levels for the majority of genes were highly correlated, but with a subset of the genes being differentially expressed (Fig. 3B; supplementary material Table S1). After counting only those transcripts altered in expression by more than 1.5-fold (with confidence values ≥70%), we identified 228 genes that were increased in expression and another 1115 genes that were decreased in expression in *Insm1^+/−^* versus *Insm1^−/−^* samples. Gene ontology analysis revealed that the majority of the more highly expressed transcripts in the *Insm1^+/−^* cells are involved in secretory cell functions, including vesicular traffic, protein processing and degradation. By contrast, the transcripts that were more highly expressed in the knockout cells were grouped into broader categories, including cytoskeleton remodeling, transcriptional regulation and components of developmental, differentiation and morphogenesis pathways (Fig. 3C; supplementary material Fig. S5).

Genes most dependent on *Insm1* for expression include those encoding pancreatic hormones, especially *Ins1* and *Ins2*, as well as components of the hormone secretory machinery such as *Chgb*, *Scg3*, *Snap25*, *Nnat* and *Vatl1*. Consistent with our immunohistochemical findings, *Ppy* was also more highly expressed in the *Insm1^−/−^* cells, as was *Cartpt*, which encodes a neuropeptide. In the absence of *Insm1* there was also a reduction in the expression of several transmembrane channels and transporters (*Kctd12b*, *Slc2a2*, *Slc30a8*, *Cacna1a*), gap junction proteins (*Gjd2*), a calcium-sensing protein (*Hpca*), several extracellular matrix proteins (*Cryba2*, *Muc4*, *Spock2*), and other less well characterized transmembrane proteins (*Tmem215*, *Lrp11*, *Amigo2*). *Insm1^−/−^* cells showed an increase in the expression of receptors previously associated with different progenitor cell types (*Tspan8*, *Prom1*, *Itgam*, *Cd59a*, *Lgr4*) and neural progenitor cells (*Nrp2*, *Pmp22*, *Fat4*, *Gfra1*, *Lamc1*). Interestingly, among the nucleic acid-binding proteins in *Insm1^+/−^* cells, there was a marked increase in the expression of genes encoding RNA-processing proteins such as *Elavl4*, *Celf3* and *Rod1*, all of which have been shown to be involved in alternative RNA splicing (Chapple et al., 2007; Spellman et al., 2007; Ince-Dunn et al., 2012). Genes for DNA-binding proteins, such as *Fev*, *St18*, *Ripply3*, *Lmo3* and *Jazf1*, were upregulated to a lesser extent. Conversely, *Insm1^−/−^* cells exhibited a marked increase in the expression of transcription factors that play important roles in the maintenance of progenitor cells (*Bhlhe22*, *Rest*, *Nr5a2*, *Hes1*, *Acsl1*, *Foxp2*, *Sox6*). Markers of EMT (*Zeb2*, *Mmp14*, *Cldn11*, *Cdh2*, *Vim*, *Cdh11*, *Fn*) and components of Notch signaling pathway (*Hes1*, *Hes5*, *Hey1*, *Dll1*, *Notch2*, *Notch1*) were also increased in the *Insm1^−/−^* progenitor cells. Similarly, we observed an upregulation in genes involved in other signaling pathways controlling cell progenitor states, such as TGF/BMP (*Smad3*, *Bmpr1b*, *Tgfb2*, *Bmp1*), FGF (*Fgfr1*, *Fgfr4*) and IGF (*Igfbpl1*, *Igfbp5*). Several genes involved in cell proliferation (*Cdkn1c*, *Bcl2*, *Mertk*, *Anxa2*, *Ccnd1*) were increased in *Insm1^−/−^* cells, whereas others (*Cdkn1b*, *Manf* and *Akt3*) were more highly expressed in the *Insm1^+/−^* cells. *Insm1^+/−^* cells also showed greater expression of genes involved in cytoskeleton reorganization and cell migration (*Gng12*, *Pak3*, *Filip1*), vascularization-promoting growth factors (*Pgf*, *Vegfa*) and a β-cell-specific MPK8 scaffold protein (*Mapk8ip2*).

We performed chromatin immunoprecipitation (ChIP) analysis of the Insm1-regulated genes *Rest*, *Cdkn1c*, *Cdkn1b* and *Ripply3*, all of which contain putative Insm1 binding sites within their promoters based on the JASPAR database (Mathelier et al., 2014) (supplementary material Table S2). All genes showed enrichment that was similar or greater than that of the *Insm1* promoter itself, which was previously shown to be downregulated by Insm1 (Breslin et al., 2002) (supplementary material Fig. S6).

### Differential expression of alternatively spliced transcripts of *Neurog3*

**Fig. 4.**
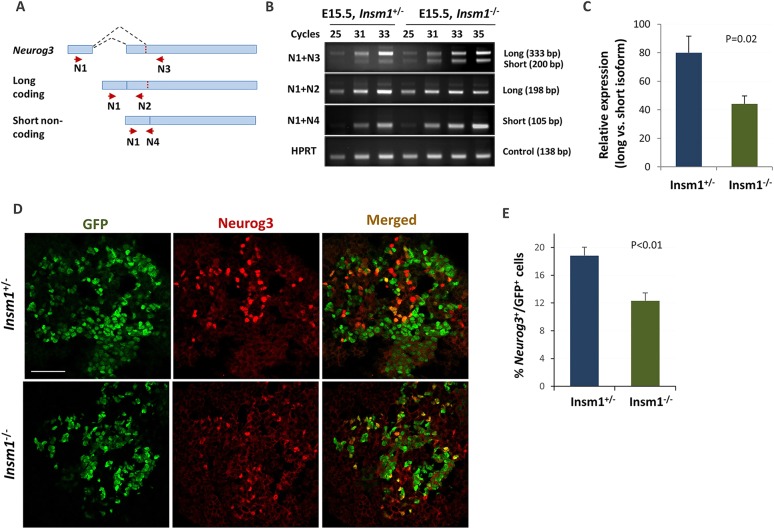
***Neurog3* is alternatively spliced in endocrine progenitor cells.** (A) *Neurog3* gene and mRNA splicing isoforms. Primer pair N1+N3 is designed to amplify both isoforms, N1+N2 the long coding isoform, and N1+N4 the short non-coding isoform. (B) Semi-quantitative PCR analysis for *Neurog3* mRNA isoforms. The proportion of the short non-coding isoform is higher in *Insm1^−/−^* than in *Insm1^+/−^* cells. (C) Quantification of relative expression of the *Neurog3* long versus short isoforms by real-time PCR. (D) Immunofluorescence labeling of pancreatic tissues from *Insm1^+/−^* and *Insm1^−/−^* mice at E15.5 with anti-GFP antibodies (green) and anti-Neurog3 antibodies (red). (E) The percentage of Neurog3-positive cells among GFP-positive cells is decreased in *Insm1^−/−^* pancreas, implying changes in Neurog3 expression on a protein level. Error bars indicate s.e.m. (*n*≥6); *P*-values were determined by Student's *t*-test. Scale bar: 50 μm.

Since alternative RNA splicing plays an important role in the differentiation of embryonic stem cells (ESCs) and the specification of many cell lineages (Fu et al., 2009; Grabowski, 2011; Llorian and Smith, 2011), and our results showed a strong *Insm1*-dependent increase in the expression of many RNA-binding splicing factors, we examined the datasets for splice junctions for a number of pro-endocrine genes. Interestingly, *Neurog3* mRNA was found to be alternatively spliced in endocrine progenitors at E15.5, with two splice variants present – a long coding and a short non-coding, with the shorter variant having about twice as many sequence tags in *Insm1^−/−^* than in *Insm1^+/−^* cells. To validate the presence of alternatively spliced *Neurog3* transcripts, we designed primers to simultaneously amplify both splicing variants or just the long or short variant (Fig. 4A), and performed semi-quantitative RT-PCR. This experiment, as well as RT-qPCR, confirmed the expression of both *Neurog3* mRNA isoforms (Fig. 4B,C; supplementary material Fig. S7), with the ratio of long to short variant expression being 2-fold higher in *Insm1^+/−^* than in *Insm1^−/−^* cells. Immunostaining of E15.5 embryos also showed a decrease in the number of GFP/Neurog3 co-expressing cells (19% in *Insm1*^+/−^ compared with 12% in *Insm1*^−/−^ tissues; Fig. 4D,E), as might be expected if there was a reduction in Neurog3 protein levels.

### Validation of RNA-Seq results by RT-qPCR and further characterization of novel genes

To further validate and quantify the differential expression of selected genes, we performed RT-qPCR using 96-well TaqMan array cards that were designed to detect genes showing higher expression in *Insm1^+/−^* versus *Insm1^−/−^* cells as well as some known pancreatic endocrine genes as a reference (supplementary material Table S3). Cells were isolated at E15.5 and E18.5, with the later time point serving to better detect changes occurring as the endocrine progenitor cells became more differentiated. This analysis confirmed the upregulation or downregulation of all these genes at E15.5 (supplementary material Fig. S8A) and allowed us to distinguish between those required for later stages of endocrine differentiation and maturation (upregulated in *Insm1^+/−^* versus *Insm1^−/−^* samples at E18.5, e.g. *Mnx1*, *Mafa*, *Ripply3* and *Fev*) and those active at earlier stages of endocrine specification (downregulated in *Insm1^+/−^* versus *Insm1^−/−^* samples at E18.5, e.g. *Neurod1*, *Runx1t1*, *Pax6* and *Pax4*). The expression of some of these genes in pancreatic endocrine cells was confirmed by examining *in situ* RNA hybridization images in the EUREXPRESS database obtained from E14.5 mouse embryos (Diez-Roux et al., 2011) (supplementary material Fig. S8B). In addition, we performed staining with antibodies against surface proteins, such as cadherin Celsr3, adhesion molecule Amigo2, and low-density lipoprotein receptor-related protein Lrp11 (supplementary material Fig. S9), and found preferential staining in each case for pre-endocrine cells.

### Temporal expression profiling of endocrine populations expressing high and low levels of Pdx1

**Fig. 5.**
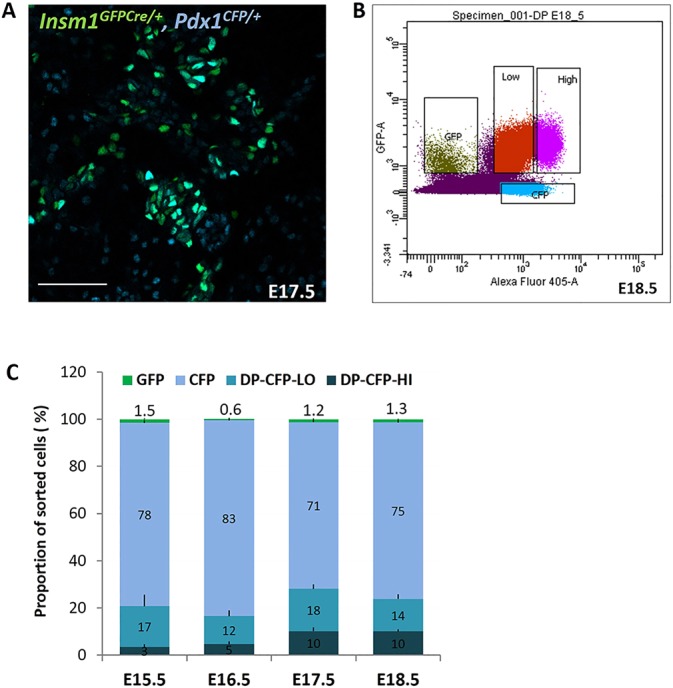
**Isolation of Insm1-positive endocrine cell populations expressing high (*Insm1*/*Pdx1*-HI) and low (*Insm1*/*Pdx1*-LO) levels of Pdx1.** (A) Confocal image of direct fluorescence in a pancreas from animals that were doubly heterozygous for both the *Insm1^GFPCre^* and *Pdx1^CFP^* alleles. GFP and CFP expression marks pre-endocrine cells, with different levels of CFP marking heterogeneous endocrine populations expressing *Pdx1* at high and low levels. Based on the known expression patterns of *Insm1* and *Pdx1* (Guz et al., 1995; Gu et al., 2002; Mellitzer et al., 2006), the perdurance of CFP for 46 h, and immunohistochemical staining of pancreatic tissues with anti-Pdx1 and anti-hormone antibodies (data not shown), these populations are thought to represent the following cells: *Pdx1*-LO, predominantly acinar cells with a small proportion of ductal cells; *Insm1*/*Pdx1*-HI, endocrine cells including mostly β-cells but also with some δ-cells; *Insm1*/*Pdx1*-LO, endocrine cells including uncommitted endocrine cells and other non-β-cells; and *Insm1*-positive neural cells of non-pancreatic origin. (B) FACS profile of pancreatic populations sorted from *Insm1^GFPCre/+^;Pdx1^CFP/+^* animals. Four distinct populations are sorted: GFP positive, CFP positive and two GFP/CFP double-positive populations expressing high (*Pdx1*-HI) and low (*Pdx1*-LO) levels of CFP. (C) Temporal changes in the relative proportions of the different sorted pancreatic cell populations. Scale bar: 50 μm.

Beginning at ∼E14.5 during pancreas development, *Pdx1* is expressed at higher levels in β-cells than in non-β-cells (Ohlsson et al., 1993). Thus, to identify *Insm1*-regulated genes that might be specifically important for β-cell development and function we used a *Pdx1^CFP^* allele (Potter et al., 2011) to isolate populations of *Insm1*-positive endocrine cells that express either high (HI) or low (LO) levels of *Pdx1* at time points from E15.5 to E18.5. Confocal imaging of direct fluorescence confirmed that *Insm1^GFPCre/+^;Pdx1^CFP/+^* mice expressed both GFP and CFP in endocrine cells, with different levels of CFP expression marking endocrine subpopulations (Fig. 5A), which we were able to separate by FACS (Fig. 5B). This allowed us to assess changes in the relative proportions of different cell types during pancreas development over a 4 day interval (Fig. 5C). *Insm1*/*Pdx1* double-positive endocrine cells comprised ∼20-30% of total sorted cells, with the greatest increase in cell numbers occurring between E16.5 and E17.5. As expected, the proportion of *Pdx1*-HI cells in the double-positive cell populations increased between E15.5 and E18.5, mirroring the increase in β-cells during pancreas development.

To explore the differences between the *Insm1*/*Pdx1*-HI and *Insm1*/*Pdx1*-LO pre-endocrine cell populations as a function of time, we performed both RT-qPCR and RNA-Seq analysis. Pairwise comparison of RNA-Seq results between these cell populations at E15.5 and E18.5 revealed an increase in the expression of a number of genes important for β-cell specification (supplementary material Tables S4 and S5). Gene ontology analysis showed that most of the *Insm1*/*Pdx1*-HI enriched genes are involved in hormone secretion as well as ion transport and glucose metabolism (supplementary material Fig. S10A,B). Ninety-three genes were commonly upregulated in *Insm1*/*Pdx1*-HI versus *Insm1*/*Pdx1*-LO cells at E15.5 and E18.5. Correlation analysis showed that many of these genes have similar differential expression levels and many are known to be important for β-cell function (supplementary material Fig. S10C).

RT-qPCR analysis of pre-endocrine cell populations from E15.5 to E18.5 allowed hierarchical clustering based on relative levels of expression of the genes in the *Insm1*/*Pdx1*-HI versus *Insm1*/*Pdx1*-LO cells (supplementary material Fig. S11). Genes expressed at higher levels in the *Insm1*/*Pdx1*-LO cells included the α-cell transcription factor *Arx*, the hormones *Glc* and *Ghrl*, as well as many transcription factors known to be involved in early endocrine specification, such as *Neurod1*, *Neurog3*, *Isl1* and *Pax4*. These results confirmed our prediction that the *Insm1*/*Pdx1*-LO population consists mostly of non-β-cells and non-committed pre-endocrine cells. Interestingly, somatostatin and *Ppy* were expressed at a higher level in the *Insm1*/*Pdx1*-HI population, especially at E15.5. Other genes that were more highly expressed in the *Insm1*/*Pdx1*-HI cells included known β-cell genes and transcription factors such as *Ins2*, *Glut2*, *Mafa*, *Mnx1* and *Nkx6.1*. This population also contained the transcriptional regulators *Mafg* and *Ripply3* and the signaling molecule *Igfbp7*.

### *Ripply3* is expressed in pancreatic β-cells and contributes to their proliferation

**Fig. 6.**
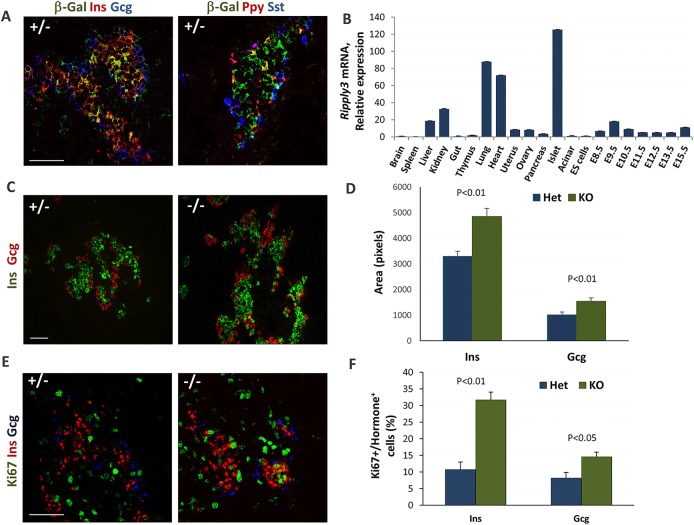
***Ripply3* is involved in the regulation of endocrine cell replication.** (A) *Ripply3* is expressed in pancreatic endocrine cells at E18.5. Immunofluorescence staining of pancreata from *Ripply3^β-Gal^* heterozygous mice with anti-β-galactosidase and antibodies against the pancreatic hormones insulin (Ins), glucagon (Gcg), pancreatic polypeptide (Ppy) and somatostatin (Sst). (B) *Ripply3* is expressed in adult pancreatic islets. RT-qPCR analysis of *Ripply3* relative expression in different tissues and during embryonic development (normalized to expression in ESCs). (C) Immunofluorescence staining for insulin and glucagon for representative sections of *Ripply3^β-Gal^* heterozygous and homozygous mice. The hormone-positive endocrine area appeared to be expanded in *Ripply3* knockout animals. (D) Quantification of total insulin-positive and glucagon-positive areas in *Ripply3^β-Gal^* heterozygous and knockout animals. Both areas are significantly increased in knockout animals. (E) Immunofluorescence staining for insulin, glucagon and Ki67 for representative sections of *Ripply3^β-Gal^* heterozygous and homozygous mice. (F) The percentage of Ki67-positive cells among total insulin-positive or glucagon-positive cells shows an increase in the proliferation of endocrine cells in *Ripply3* knockout animals. Error bars indicate s.e.m. (*n*≥6); *P*-values were determined by Student's *t*-test. Scale bars: 50 μm.

Since the function of *Ripply3*, a gene we found to be both upregulated by *Insm1* and enriched in *Insm1*/*Pdx1*-HI cell populations, had not been previously explored in pancreas, we analyzed mouse pancreatic tissues from *Ripply3^β-Gal^* mice, which express β-galactosidase instead of *Ripply3* (Okubo et al., 2011). Co-staining for β-galactosidase, chromogranin A and islet hormones confirmed that *Ripply3* is expressed in nascent endocrine cells, with higher levels detected in β-cells at E18.5 (Fig. 6A; supplementary material Fig. S12A). RT-qPCR on embryonic and adult tissues showed that *Ripply3* is strongly expressed in heart and lung, and to a lesser degree in liver and kidney. In the adult pancreas the expression of *Ripply3* was highest in islets (Fig. 6B). Based on RNA-Seq profiling of distinct pancreatic developmental populations, *Ripply3* is expressed in early endoderm at E8.0, with lower levels detected in pancreatic endoderm and MPCs, and increased expression in *Neurog3*-positive endocrine progenitor cells at E15.5 and further in nascent β-cells at E16.5 (supplementary material Fig. S12B). Although pancreatic endocrine cell types were present at E18.5 in *Rippy3^β-Gal^* heterozygous and knockout mice, both β-cell and α-cell areas were increased (Fig. 6C,D; supplementary material Fig. S12C). In addition, there was a notable (although not statistically significant) increase in somatostatin-positive and Ppy-positive cell area (data not shown). Co-staining of insulin-expressing and glucagon-expressing cells for Ki67 indicated that the proliferation of β-cells was increased in the *Ripply3* knockout pancreata (Fig. 6E,F). The increase in proliferation was not accompanied by the loss of differentiated β-cell phenotype since knockout tissues expressed levels of the maturation markers Glut2, Mafa, Mafa and Nkx6.1 similar to those of the heterozygote (supplementary material Fig. S12D). TUNEL assay showed no increase in apoptosis of β-cells in *Ripply3* null animals at E18.5 (data not shown).

## DISCUSSION

### Role of *Insm1* in pancreatic pre-endocrine cell differentiation

In agreement with previously published data (Gierl et al., 2006), mice without *Insm1* have a reduced number of endocrine cells and hormone expression in these cells is either lacking or imbalanced in terms of specific endocrine cell types. Given that Insm1 has long been known to contain a SNAG repressor domain (Chiang and Ayyanathan, 2013), our finding that approximately twice as many genes in *Insm1^−/−^* than in *Insm1^+/−^* mice were increased in expression is consistent with the major role of Insm1 being as a repressor. In the absence of *Insm1*, pancreatic pre-endocrine cells display many features of earlier stage progenitor cells, including an increase in the expression of transcription factors and cell surface receptors associated with the progenitor state, EMT markers, and components of morphogenic signaling pathways (e.g. Notch, Tgf/BMP and Wnt)*.* Although EMT markers increased in *Insm1* null cells, they failed to migrate away from the ducts. This might be due to incomplete EMT, since some the genes upregulated in *Insm1^+/−^* cells are involved in actin remodeling and cell migration. Moreover, signaling molecules and extracellular matrix proteins that are expressed upon *Insm1*-dependent endocrine differentiation might be necessary for the migration and aggregation of newly differentiated endocrine cells into islets. *Insm1* null cells also have decreased rates of proliferation that starts at E15.5 and is well pronounced at E18.5. This decrease was not accompanied by an increase in apoptosis, and the knockout cells appeared larger in size, a characteristic that often accompanies cell cycle arrest at G1/G2 phase (Bjorklund et al., 2006). Consistent with cell cycle arrest without apoptosis, *Insm1* null cells express more of the cell cycle inhibitor Cdkn1c and the pro-survival gene *Bcl2*. Interestingly, *Insm1*-expressing cells make more of the cell cycle inhibitor Cdkn1b (p27^Kip1^), which is involved in controlling the proliferation of newly differentiated β-cells (Georgia and Bhushan, 2006). This suggests that *Insm1* promotes a switch in the mode of cell cycle regulation from being Cdkn1c based to being Cdkn1b regulated, with both genes being directly regulated by Insm1. A similar developmental switch in cell cycle regulators has been observed during pituitary development, in which non-cycling precursors express Cdkn1c and more differentiated cells express Cdkn1b (Bilodeau et al., 2009). Although the differentiation of endocrine cells in the presence of *Insm1* is associated with an increase in proliferation, cell proliferation itself is under the control of multiple genes, including cell cycle inhibitors such as *Cdkn1b* and *Ripply3*. The complex regulation of endocrine progenitor cell proliferation is not surprising given that overexpression of *Insm1* in the developing neural cortex has been shown to decrease the proliferation of less differentiated apical progenitor cells but to increase the proliferation of more differentiated intermediate basal progenitor cells (Farkas et al., 2008).

This study also brings to light several genes that might play important but previously unrecognized roles in the formation of islet cells. Indeed, although a key function of *Insm1* might be to repress genes (e.g. *Rest*) that maintain pancreatic ductal epithelial cells in a progenitor-like state, which would be expected to promote endocrine cell differentiation, many genes were also found to be upregulated in *Insm1*-expressing cells. These include genes necessary for hormone secretion, membrane transporters and channels, extracellular matrix proteins, RNA-processing proteins and cell migration proteins. For example, *Insm1*-expressing progenitor cells are characterized by higher levels of transcription factors that are found in differentiating β-cells (*Mafa*, *Mafb*, *Mnx1*, *Vdr*, *Hmgn3*, *Fev*, *Lmx1b*), as well as novel genes that have not been studied in the pancreas (*Lmo3*, *Ripply3*, *Jazf1*, *Nhlh1*). Among the *Myt1* family, *St18*, *Myt1* and *Myt1l1* were all upregulated, suggesting that all three of these genes are expressed in differentiating endocrine cells, contrary to a previous report (Gu et al., 2004). Considering that many of the *Insm1*-regulated transcription factors have also been shown to play key roles during neural development, and that neural and islet cells share many phenotypic properties, these genes probably also contribute to endocrine development. Interestingly, one such gene, the transcription factor *Jazf1*, is associated with the development of type 2 diabetes (Zeggini et al., 2008).

### Identification of novel endocrine surface markers

This study revealed a number of surface proteins that mark early endocrine cells. For example, mRNAs for *Amigo2*, *Celsr3* and *Lrp11* were strongly upregulated in *Insm1*-positive cells, and immunostaining for the encoded proteins confirmed their localization along the membrane of endocrine cells. Amigo2 showed the strongest endocrine-specific staining pattern and may function in cell adhesion and migration (Rabenau et al., 2004). Celsr3, the cadherin associated with planar polarity, displayed localized membrane staining and was recently shown to promote endocrine cell differentiation (Tissir and Goffinet, 2006; Cortijo et al., 2012)*.* Another early endocrine cell marker validated by our study is *Lrp11*, as was previously suggested by Hald et al. (2012). Interestingly, although Lrp11 is present in all endocrine cells, it seems to be most highly expressed in δ-cells.

### *Neurog3* is alternatively spliced in endocrine progenitor cells

RNA-Seq revealed that ∼30% of the differentially expressed genes had two or more differentially expressed RNA variants (data not shown). This suggests that the *Insm1*-dependent differentiation program might also function at a post-transcriptional level by promoting endocrine lineage-specific alternative splicing. Although the mechanisms involved in this regulation are unclear, this could occur through the activation of genes encoding RNA-binding proteins, such as *Elavl4*, *Celf3* and *Rod1*. All of these transcripts were strongly upregulated in *Insm1*-positve cells and so could be important in regulating splicing changes during endocrine differentiation. In particular, we found that *Neurog3* mRNA is alternatively spliced in endocrine progenitor cells, with a long coding and short non-coding isoform present. The latter is formed through splicing to the alternative splice acceptor site located in the second exon of *Neurog3* (supplementary material Fig. S7). Interestingly, the ratio of the *Neurog3* coding to non-coding isoform was higher in *Insm1*-positive cells, suggesting that alternative splicing of the *Neurog3* pre-mRNA in an *Insm1*-dependent manner provides a positive-feedback mechanism for maintaining *Neurog3* expression during development.

### Transcriptional profiling of subpopulations of pre-endocrine cells

Combinatorial FACS using two or more fluorescent reporter alleles is a powerful means of isolating specific progenitor cell subpopulations. For this reason, we also made use of a recently derived *Pdx1^CFP^* fluorescent reporter allele to explore the differences in gene expression between developing endocrine progenitor cells that express either high or low levels of *Pdx1*. It is well known that *Pdx1* marks all endocrine cells (Herrera, 2000; Gu et al., 2002) and that developing β-cells, as well as a small fraction of δ-cells, express high levels of *Pdx1* (Leonard et al., 1993; Ohlsson et al., 1993; Guz et al., 1995). As expected, *Insm1*/*Pdx1-*HI cells express more β-cell-specific genes, such as *Ins2*, *Slc2a2* and *Mafa*. However, we were surprised to find that this cell population (at E15.5) also contains many somatostatin and *Ppy* transcripts. This suggests that the *Insm1*/*Pdx1-*HI cell population might contain a common β/δ-progenitor, as well as newly formed δ-cells, the existence of which has been predicted by *Pax4* and *Arx* knockout studies (Collombat et al., 2003, 2005). Our data are also consistent with previous lineage-tracing studies that have shown that a *Ppy*-*Cre* transgene marks both β-cells and Ppy-producing cells (PP-cells) (Herrera et al., 1994; Herrera, 2000), a finding that suggests that PP-cells and a common β/δ-cell might share common a progenitor, or that Ppy is expressed in the common β/δ-cell progenitor. Analysis of differential expression between *Insm1*/*Pdx1-*HI and *Insm1*/*Pdx1-*LO endocrine subpopulations allowed us to define *Insm1*-dependent genes that are enriched in *Pdx1*-HI populations and therefore potentially important for specific functions of β-cells and/or δ-cells.

### *Ripply3* as a downstream modulator of endocrine cell proliferation

In this study we discovered that *Ripply3* is also expressed in endocrine progenitor cell populations and that Insm1 binds to the *Ripply3* promoter. Ripply3 belongs to a family of related proteins, the other two members of which, Ripply1 and Ripply2, are necessary for the proper formation of somite boundaries and function by antagonizing T-box transcription factor activity during development (Kawamura et al., 2005; Hitachi et al., 2009; Takahashi et al., 2010). Ripply3 has also been shown to regulate pharyngeal apparatus development through suppression of *Tbx1* activity (Okubo et al., 2011). The importance of other Ripply gene family members prompted us to explore the role of *Ripply3* in pancreas development. Although our analysis did not reveal any defects in endocrine cell differentiation in *Ripply3* null pancreatic tissues, we observed that both β-cell and α-cell areas and proliferation were increased.

The increase in proliferation in *Ripply3* null tissues suggests that, similar to *Cdkn1b*, *Ripply3* might control the proliferation of newly committed β-cells in order to allow them to properly differentiate. The mechanism by which Ripply3 functions in pre-endocrine cells is unclear. As mentioned above, members of this class of proteins have been shown to bind and suppress the activity of T-box transcription factors. Previous reports (Begum and Papaioannou, 2011; Oropez and Horb, 2012) have shown that *Tbx2* is expressed in pre-endocrine cells, and our current findings indicate that it is upregulated in the absence of *Insm1*. Thus, given that Tbx2 was previously shown to promote cell proliferation (Vance et al., 2005), Ripply3 might function by antagonizing Tbx2 activity in differentiating endocrine cells. In any case, the functional interactions between *Ripply3* and *Tbx2*, and how each gene might contribute to the growing network of genes that promote and/or regulate the proliferation of pancreatic endocrine progenitor cells, merit additional study.

### Conclusions

**Fig. 7.**
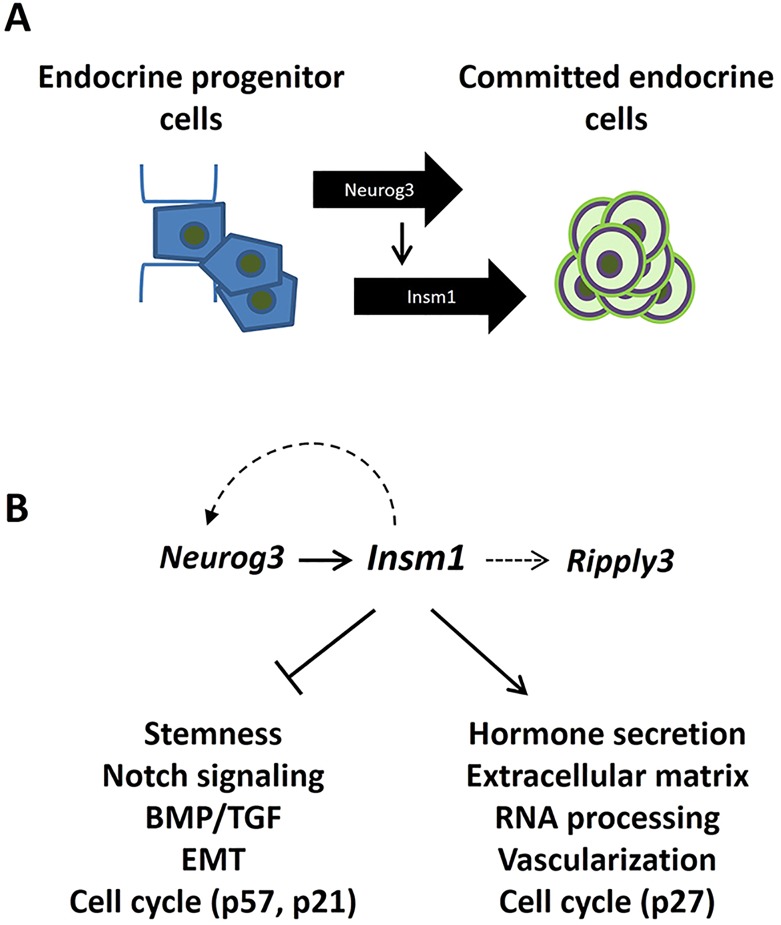
**Model for the role of *Insm1* in endocrine progenitor cells.** (A) The *Insm1* transcriptional program regulates a progression of pre-endocrine cells from *Neurog3*-positive endocrine progenitors residing in a duct to islet-bound committed endocrine cells. (B) Endocrine progenitor cells are characterized by Notch signaling and other stemness-controlling pathways. As endocrine cell differentiation occurs, the proliferation of these cells slows as they exit the cell cycle and delaminate from the duct through the activation of the EMT program. *Insm1* promotes these changes by repressing progenitor cell functions while simultaneously activating genes involved in hormone secretion, formation of extracellular matrix, cell migration, and promotion of alternative splicing and vascularization. The *Insm1*-dependent endocrine differentiation program is accompanied by an increase in the coding isoform of *Neurog3* and expression of *Ripply3*, a gene we have shown to regulate β-cell proliferation.

These studies provide valuable new insights into the role that *Insm1* plays in developing pancreatic endocrine cells (as summarized in the model shown in Fig. 7). We expect that the RNA-Seq datasets, besides identifying many genes that are affected in expression by *Insm1*, will serve as a platform for further discoveries, especially as more comprehensive models of the gene regulatory network in endocrine cells are built.

## MATERIALS AND METHODS

### Antibodies and primers

Antibodies employed in immunohistochemistry and ChIP and primers used for genotyping, qPCR and ChIP analysis are described in the Methods in the supplementary material.

### Mouse lines

The *Insm1^GFPCre^* allele was made using a sequential gene targeting and RMCE strategy using methods and vectors that have been described previously (Chen et al., 2011). *Insm1^GFPCre^* and *Pdx1^CFP^* mice (Potter et al., 2011) were maintained on a CD1 outbred background (90% or higher) to extend the survival of the embryos, as previously described (Gierl et al., 2006). Approximately 10% of the *Insm1* homozygous null pups survived to E18.5, consistent with the results of Gierl et al. (Gierl et al., 2006), with all other embryos dying between E15.5 and E18.5. *Ripply3^β-Gal^* mice were of mixed C57BL6/129SV background. All animal procedures and husbandry protocols were approved by the Vanderbilt University Institutional Animal Care and Use Committee.

### Embryo isolation and imaging, tissue dissociation and FACS

Timed matings were carried out by designating noon on the day vaginal plugs were identified as E0.5. Embryos at different stages were obtained by dissection and imaged by direct fluorescence visualization using a Leica MZ 16 FA stereoscope equipped with a QImaging RETIGA 4000R camera. For FACS, embryos were isolated at E15.5-18.5 and initially genotyped by direct fluorescence. Pancreata were dissected in cold PBS and dissociated with Accumax solution (Sigma-Aldrich) containing 5 μg/ml DNase I (Sigma-Aldrich) at 37°C for 1 h with periodic resuspension. Quenching solution [L15 medium, 10 mM Hepes (pH 7.0), 1 mg/ml bovine serum albumin (Life Technologies), 5 μg/ml DNase I] was added to stop the reaction. The cell suspension was filtered through a 35 μm filter into FACS tubes, washed with quenching solution and resuspended in 0.5 ml quenching solution containing 7AAD viability dye (1:1000; Life Technologies). Fluorescent cells were sorted directly into Trizol LS reagent (Life Technologies) containing 40 μg/ml mussel glycogen (Sigma-Aldrich) using a FACSAria I cell sorter (BD Biosciences).

### RNA isolation, library construction and RNA-Seq

Three independent RNA isolates from each genotype were used for cDNA amplification and sequencing. Cells from multiple embryos were pooled to obtain a sufficient amount of RNA for analysis. The methods used to isolate RNA, validate integrity and purity, amplify cDNA and make libraries have been described previously (Choi et al., 2012). Single-end sequencing (110 bp) was performed on an Illumina HiSeq2000 genome analyzer. Read alignment to the mouse genome (mm9) was performed using RNA-Seq Unified Mapper (RUM) (Grant et al., 2011). Expression level was quantified as reads per kilobase of gene model per million mapped reads (RPKM). Genome alignment of sequencing data yielded total mapped reads in a range from 30 to 47 million per sample, from which 66-70% of reads were uniquely mapped. RPKM values of the different samples were highly correlated between replicates (R^2^=0.99). Differential expression analysis was performed using Patterns of Gene Expression (PaGE) software (Grant et al., 2005). Summaries of the differential expression analyses are provided in supplementary material Tables S1, S3 and S4. Gene ontology analysis was performed using DAVID Bioinformatics Resources v.6.7 (Huang et al., 2009). RNA-Seq data are available at http://genomics.betacell.org (study numbers 4294 and 4674) and are also deposited at ArrayExpress (accession number E-MTAB-1929).

### RT-qPCR analysis

Ninety-six gene custom TaqMan Low-Density Array (TLDA; Life Technologies) cards were used for validation of gene expression. The TaqMan probe and primer sets were selected from predesigned TaqMan gene expression assays (Life Technologies). Three independent RNA isolates for each genotype or developmental time point were assayed. cDNA was prepared from 30 μg total RNA using a high-capacity cDNA Archive Kit (Life Technologies) and divided between two ports of the TLDA cards. PCR was performed with an ABI 7900HT real-time PCR system (Life Technologies) and amplification data were analyzed using Sequence Detection System version 2.1 (Life Technologies) and Excel software (Microsoft). 18S ribosomal RNA and *Gapdh* were used as endogenous housekeeping controls for normalization, and the comparative Ct method was used to calculate relative fold expression by 2^−ΔΔCt^ (supplementary material Table S2). Hierarchical clustering analysis of the data was performed using GenePattern software (Reich et al., 2006). The clustering was performed on relative expression levels (log_10_) using Euclidean distance and pairwise average linkage. The Hierarchical Clustering Viewer from GenePattern was used for graphical representation, with coloring relative for each gene row. For analysis of *Neurog3* alternative splicing variants, 1 ng amplified cDNA (used for RNA-Seq) was amplified using either real-time PCR with Power SYBR Green PCR Master Mix (Life Technologies) or semi-quantitative PCR using Phusion Taq (New England Biolabs).

### Immunohistochemistry

Immunofluorescence staining of frozen sections was essentially as previously described (Burlison et al., 2008). Ductal cells were stained with biotinylated *Dolichos biflorus* agglutinin (DBA; Vector Laboratories) followed by streptavidin-Cy5 conjugate (Vector Laboratories). Coverslips were mounted using ProLong Gold antifade reagent with DAPI (Life Technologies). Fluorescence images were acquired using an Axioplan2 microscope (Zeiss) with 20× and 40× objectives and QImaging RETIGA EXi camera. Confocal microscopy images were acquired using an LSM 510 Meta microscope and imaging software (Zeiss). Images taken with different color channels were merged using Photoshop (Adobe Systems).

### Morphometric analysis

The fractions of GFP-positive or β-galactosidase-positive endocrine cells co-expressing other proteins were calculated by manual cell counting on step sections (50 μm apart) of pancreatic tissues from at least six different animals of the same age and genotype. At least 500 cells were counted for each phenotype in each experiment. The percentage of GFP-positive cells adjacent to ducts was calculated based on the number of these cells in contact with DBA-positive cells. The cell area of GFP-positive endocrine cells was measured using ImageJ (NIH) software to outline E-cadherin-positive cell borders using a total of 1000 cells from three different animals of each phenotype. The insulin-positive and glucagon-positive areas of *Ripply3* heterozygous and mutant animals were measured using every fifth section from three different animals of the same phenotype.

### ChIP

Pancreata were dissected from E15.5 wild-type embryos, cross-linked with 1% formaldehyde in PBS for 8 min, quenched with 125 mM glycine and washed with PBS. Tissues were lysed in 100 μl lysis buffer [50 mM Tris-HCl (pH 8.0), 10 mM EDTA, 1% SDS, 1× protease inhibitor mix (Sigma), 1 mM PMSF], sonicated (Bioraptor, Diagenode) and lysates diluted to 1 ml with RIPA buffer [10 mM Tris-HCl (pH 7.5), 140 mM NaCl, 1 mM EDTA, 0.5 mM EGTA, 1% Triton X-100, 0.1% (w/v) SDS, 1× protease inhibitor mix, PMSF]. 20 μg sonicated chromatin was incubated with 8 μg antibody coupled to IgG magnetic beads (Cell Signaling Technology) overnight at 4°C. The beads were washed five times with RIPA buffer and once with TE buffer [10 mM Tris (pH 8.0), 1 mM EDTA]. After washing, bound DNA was eluted at 65°C in elution buffer [20 mM Tris-HCl (pH 7.5), 5 mM EDTA, 50 mM NaCl, 1% SDS, 50 μg/ml proteinase K] for 2 h. After cross-linking reversal, the immunoprecipitated DNA was purified by phenol-chloroform extraction and ethanol precipitation and resuspended in 30 μl TE buffer. Enrichment at target promoters was determined by real-time PCR with Power SYBR Green PCR Master Mix. Relative fold enrichment at different sites was calculated by the 2^−ΔΔ^^CT^ method from five different ChIP experiments.

## Supplementary Material

Supplementary Material
