# Supplementary material for: Insm1 promotes endocrine cell differentiation by modulating the expression of a network of genes that includes Neurog3 and Ripply3
Source: Development. 2014 Aug;141(15):2939–49. doi: 10.1242/dev.104810 (PMC4197673; doi:10.1242/dev.104810)
Supplement: Supplementary Material [file supp_141_15_2939__index.html]

Insm1 promotes endocrine cell differentiation by modulating the expression of a network of genes that includes Neurog3 and Ripply3 — Supplementary Material 

# *Insm1* promotes endocrine cell differentiation by modulating the expression of a network of genes that includes *Neurog3* and *Ripply3*

## DEV104810 Supplementary Material

**Files in this Data Supplement:**

- **Supplementary Material**
