# Supplementary material for: Insm1 promotes endocrine cell differentiation by modulating the expression of a network of genes that includes Neurog3 and Ripply3
Source: Development. 2014 Aug;141(15):2939–49. doi: 10.1242/dev.104810 (PMC4197673; doi:10.1242/dev.104810)
Supplement: Supplementary Material [file supp_141.15.2939_DEV104810.pdf]

Supplementary Methods

Primers. Genotyping of the *Insm1*^{GFPCre} allele was performed using primers IA3: 5′-GCCCTTGTACAACCGACAGCTCT and IA4: 5′-GCCCTTGTACAACCGACAGCTCT, which amplify a 423 bp fragment from the wild-type allele and a 362 bp fragment from the *Insm1*^{GFPCre} allele. Genotyping of the *Pdx1*^{CFP} allele was performed using primers IA3: 5′-GCCCTTGTACAACCGACAGCTCT and IA4: 5′-GCCCTTGTACAACCGACAGCTCT, which amplify a 423 bp fragment from the wild-type allele and a 362 bp fragment from the *Insm1*^{GFPCre} allele. Genotyping of the *Ripply3*$^{\beta\text{-}Gal}$ allele was performed using primers Rpy3-geF1: 5′-AACCTGAGATCGACTACTGC-3′ and Rpy3-geR1: ATCCCTTAAGGTCTGTCTGC, which amplify a 350 bp fragment from the wild-type allele; and lacZ-F1: 5′-TGTTTTGACCGCTGGGATCTGC-3′ and lacZ-R1 5′-CCAGACCAACTGGTAATGGTAGC-3′, which amplify a 550 bp fragment from the *Ripply3*$^{\beta\text{-}Gal}$ allele. For qPCR analysis of *Neurog3* alternative splicing variants the following primers were used: (N1) 5′-CAGCTATCCACTGCTGCTTG; (N2) 5′-GAATTGGAACTGAGCACTTC; (N3) 5′-GTTTGCTGAGTGCCAACTC; and (N4) 5′-CGGAGCAGTCCCTAGTTCTC. RT-qPCR for *Ripply3* gene expression was performed on 50 ng cDNA using primers 5′-TGAGTCTTGGGGAGACCAAC and 5′-AGAAATGAATGGTGGCTTGC. Gene expression was normalized using the *Hprt* gene, which was amplified with primers 5′-TACGAGGAGTCCTGTTGATGTTGC and 5′-GGGACGCAGCAACTGACATTTCTA. For ChIP analysis the following primers were used. *Insm1* promoter: 5′-GAGGAGCTGCGGACGCTCTGATTG-3′, 5′-GACGCGCGTGCGGAGCGCAGAG-3′; *Rest* promoter forward 5′-GATTAGGTAAGTTTCCCCCGAG-3′, reverse 5′-CGCACATTCCAGCACAGGAC-3′; *Cdkn1c* promoter forward 5′-CTGTGCCCAGCTCCATGGTC-3′, reverse 5′-AGAGCTCTGCGGGCGGTAAC-3′; *Cdkn1b* promoter forward 5′-

GAGCGGTCAGTCCTGGCTTC-3′, reverse 5′-GGAGGGTATGCGAGAGATTCAG-3′; *Ripply3* promoter forward 5′-GCACTGGAGAACAGGTTATTGG-3′, reverse 5′-GTGACAGCTGGGTTTACGAAG-3′; *Pck1* promoter forward 5′-CAGGCAGGGTCAAAGTTTAGTC-3′, reverse 5′- GGCAGGCCTTTGGATCATAGCC-3′.

Antibodies. For immunohistochemical staining the primary antibodies and dilutions used were as follows: chicken anti-GFP (A10262, Life Technologies), 1:500; rabbit or guinea pig anti-PDX-1 (a gift from Christopher Wright, Vanderbilt University, Nashville, TN, USA), 1:1000; guinea pig anti-INS (A0564, DACO), 1:1000; rabbit anti-GCG (AB932, EMD Millipore), 1:1000; sheep anti-SST (13-2366, American Research Products), 1:1000; rabbit anti-GHRL (19-031-30, Phoenix Pharmaceuticals), 1:1000, rabbit anti-PPY (AB15668, EMD Millipore), 1:1000; mouse anti-MKI67 (550609, BD Biosciences), 1:1000; goat anti-NEUROG3 (AB2774, BCBC Antibody Core), 1:500; mouse anti-INSM1 (AB2154, BCBC Antibody Core), 1:200; rabbit anti-LRP11 (C16508, Assay Biotech), 1:500; rabbit anti-CELSR3 (sc-99200, Santa Cruz Biotechnology), 1:100; rabbit anti-AMIGO2 (252046, Ab Biotech), 1:1000; rabbit anti-GLUT2 (07-1402, EMD Millipore), 1:1000; rabbit anti-MAFA (A300-611A, Bethyl Laboratories), 1:1000; rabbit anti-MAFB (IHC-0351, Bethyl Laboratories), 1:1000; rabbit anti-NKX6.1 (AB1069, BCBC Antibody Core), 1:1000; chicken anti-β-galactosidase (AB9361, Abcam), 1:500; rabbit anti-Chromogranin A (AB15160, Abcam), 1:1000; rabbit anti-E-cadherin (4065, Cell Signaling), 1:1000. Secondary antibodies (Life Technologies, 1:1000) were as follows: donkey anti-mouse Alexa 488 conjugated (A-21202); goat anti-chicken IgG Alexa 488 (A-11039) or 555(A-21437) conjugated; goat anti-guinea pig IgG Alexa 555 (A-21435) or 647 (A-21450) conjugated; donkey anti-rabbit IgG Alexa 555 (A-31572) or 680 (A-10043) conjugated; donkey anti-sheep IgG Alexa 555 (A-21099) conjugated. Staining with anti-

INSM1 antibodies required the use of the TSA amplification kit (PerkinElmer). ChIPs were performed using rabbit polyclonal anti-INSM1 antibodies (sc-68355X) and normal rabbit antibodies (sc-2027, Santa Cruz Biotechnology).

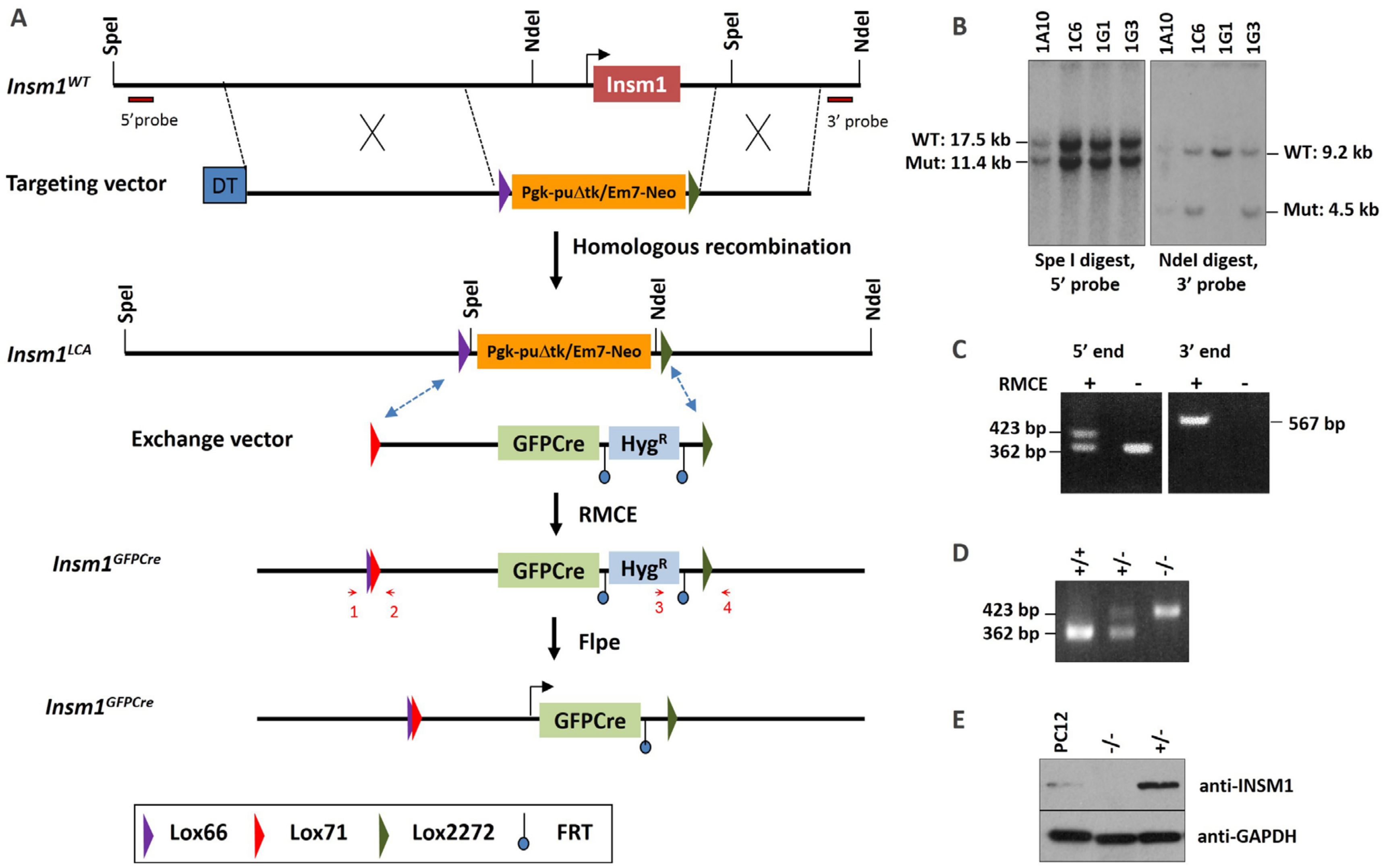

Supplementary Figure S1. Generation of *Insm1*^{GFPCre} allele. (A) Gene targeting and RMCE strategy. An *Insm*^{LCA} allele was made by gene targeting in mESCs. A 5232 bp fragment containing the *Insm1* promoter region, single exon and 3' UTR were replaced by positive-negative selection marker puroDtk surrounded by heterotypic Lox66 and Lox2272 sites. Puromycin-resistant clones were screened by Southern blotting. To screen for homologous recombination on the 5' end DNA was digested using SpeI restriction enzyme and hybridized with the 5' probe; for the 3' end screen DNA was digested with NdeI and hybridized with the 3' probe. A correctly targeted clone (1G3) containing the *Insm1*^{LCA} allele was used for RMCE to create *Insm1*^{GFPCre} allele introducing *Insm1* promoter and GFP-Cre fusion gene. Clones resistant to hygromycin were screened for cassette exchange by PCR with primers 1+2 and 3+4 to assess site specific recombination at the 5'- and 3'-ends, respectively. Clone 1G3/3B3 was used to make mice which were then crossed to animals expressing FlpE to delete the hygromycin-resistance cassette. (B) Southern blot analysis. The presence of 11.4 kb band with 5' probe and 4.5 kb band with 3' probe indicates positively targeted clones. (C) PCR analysis. Exchanged clones are characterized by double bands (423 and 325 bp) at 5' end, and a single 567 bp band at the 3' end. (D) *Insm1*^{GFPCre} mouse genotyping is done using the same primers as for 5' RMCE screen. (E) *Insm1*^{GFPCre} homozygous mice do not express INSM1 protein as confirmed by Western blot with anti-INSM1 antibodies. The PC12 cell line was used as positive control for INSM1 expression.

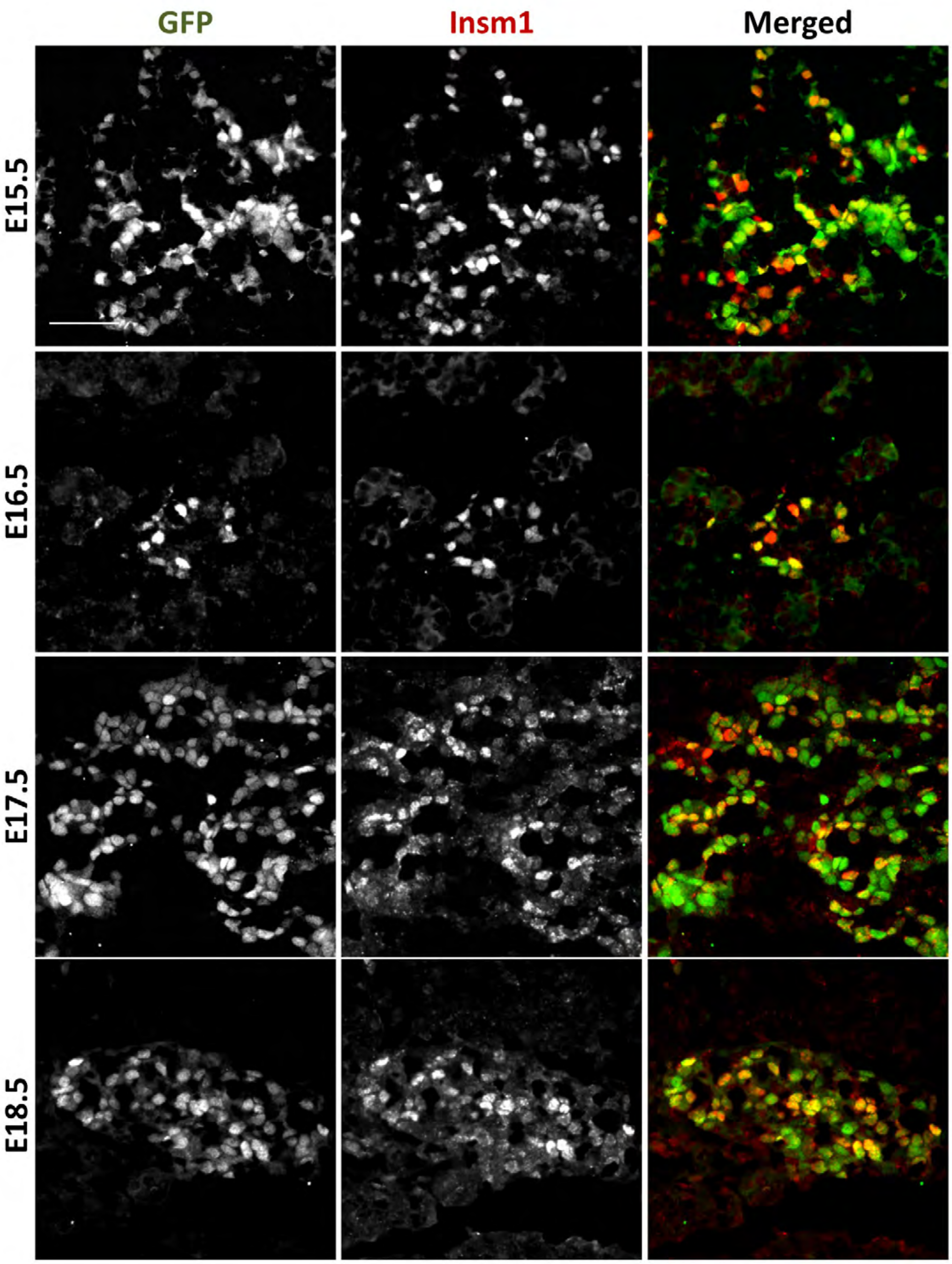

Supplementary Figure S2. GFP is co-expressed with Insm1 in *Insm1*$^{GFPCre/+}$ mice. Immunofluorescence labeling with anti-GFP (green) and anti-INSM1 (red) antibodies shows that GFP is co-expressed with *Insm1* in pancreatic endocrine cells from E15.5 to E18.5. Scale bar = 50 mm.

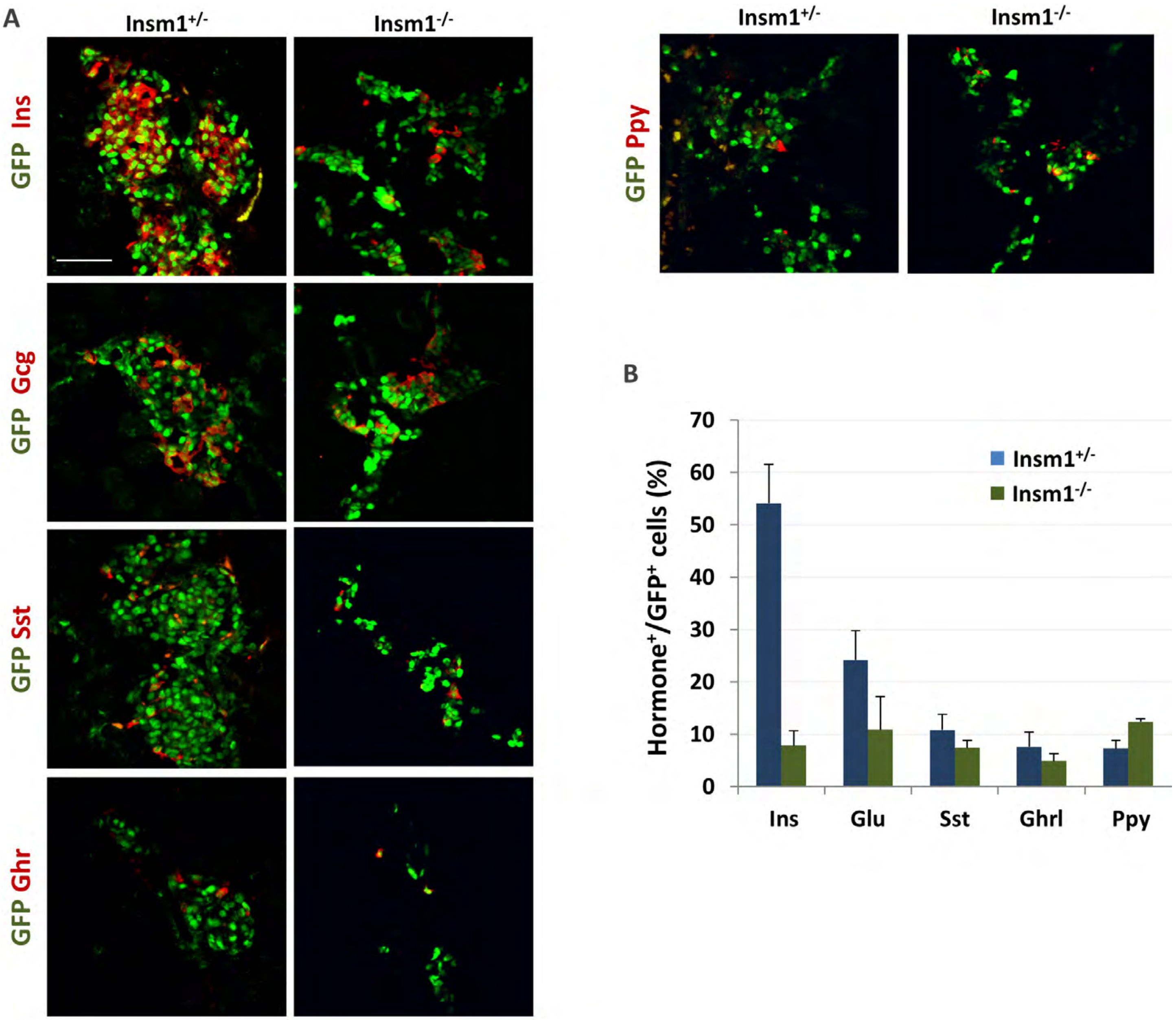

Supplementary Figure S3. Impaired differentiation of endocrine cells in *Insm1* knockout mice. (A) Immunofluorescence labeling of pancreatic tissues from *Insm1*$^{+/-}$ and *Insm1*$^{-/-}$ mice at E18.5 with anti-GFP antibodies (green) and antibodies against pancreatic hormones (red): insulin (Ins), glucagon (Gcg), somatostatin (Sst), ghrelin (Ghrl) and pancreatic polypeptide (Ppy). (B) Percentage of GFP-positive cells co-expressing different hormones at E18.5 in *Insm1*$^{+/-}$ and *Insm1*$^{-/-}$ mice ($p \leq 0.01$ for all). Scale bar = 50 mm.

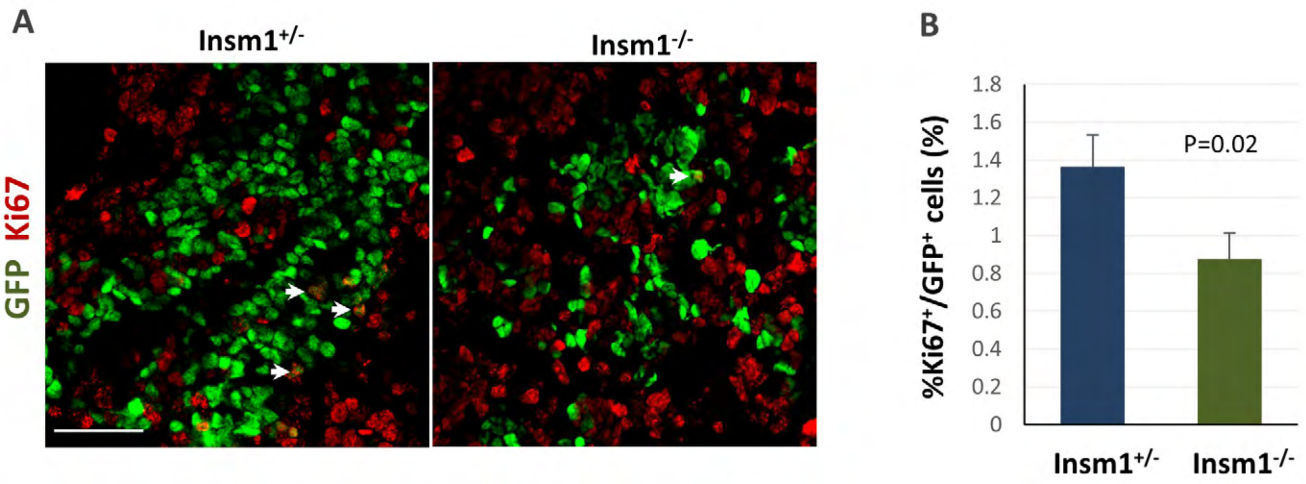

Supplementary Figure S4. Analysis of proliferation of pre-endocrine cells in *Insm1* knockout mice at E15.5. (A) Immunofluorescence labeling of pancreatic tissues from *Insm1*$^{+/-}$ and *Insm1*$^{-/-}$ mice at E15.5 with anti-GFP antibodies (green) and antibodies against cell proliferation marker Ki-67(red). (B) Percentage of Ki-67 positive cells per GFP positive cells demonstrates slight proliferation defect in *Insm1*$^{-/-}$ pancreas. ($p<0.02$). Scale bar = 50 mm.

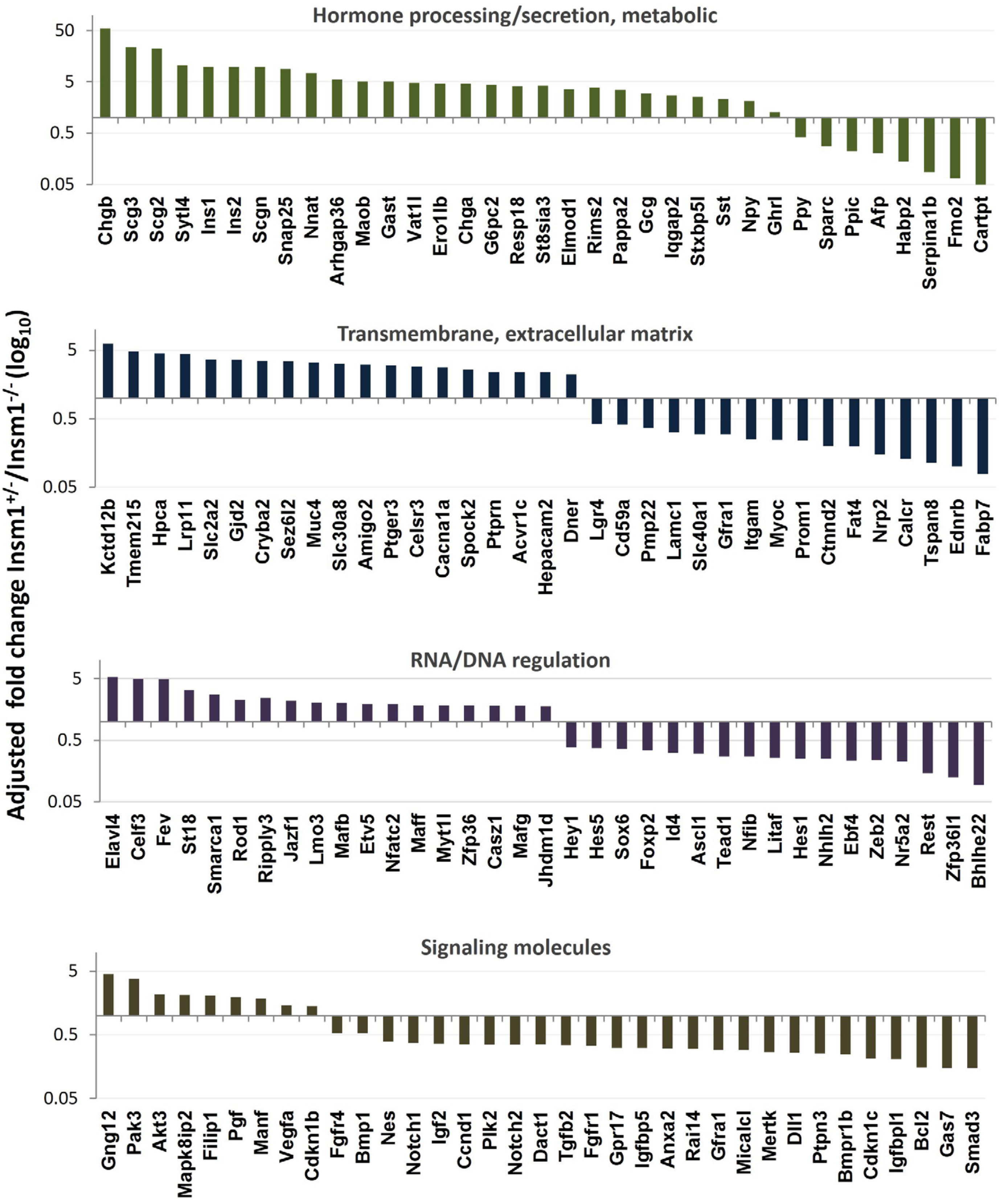

Supplementary Figure S5. Genes differentially expressed in *Insm1*$^{+/-}$ and *Insm1*$^{-/-}$ endocrine cell populations at E15.5. Representation of genes differentially expressed in *Insm1*$^{+/-}$ and *Insm1*$^{-/-}$ cell populations within different functional groups. Adjusted fold change value in *Insm1*$^{+/-}$ and *Insm1*$^{-/-}$ gene expression comparisons (an adjustment factor was added to numerator and denominator to avoid extremely small numbers in the denominator).

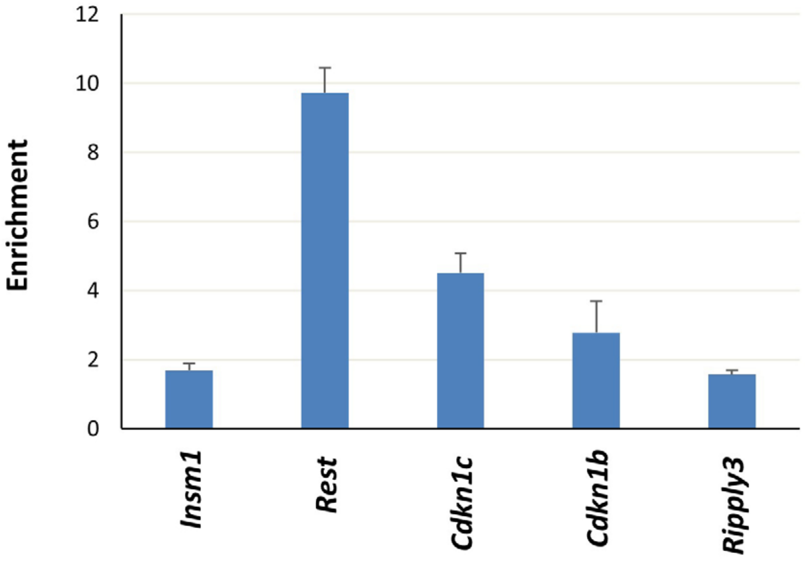

Supplementary Figure S6. ChIP analysis of Insm1 binding to the promoters of potential target genes. Chromatin immunoprecipitations with anti-INSM1 antibodies revealed enrichment at the promoter regions of *Rest*, *Cdkn1c*, *Cdkn1b* and *Ripply3* over a control *Pck1* promoter, which does not have INSM1 binding sites. *Insm1* promoter region, previously being shown to be bound by INSM1 (Breslin et al., 2002), was used as a positive control. Relative fold enrichment at different sites was calculated by $2^{-\Delta\Delta Ct}$ method where $\Delta\Delta Ct$ is (Ct target promoter (INSM1 antibody)–Ct target promoter (IgG control))-(Ct control promoter (anti-INSM1 antibody)–Ct control promoter (IgG control)). $P \leq 0.05$ for all tested promoters.

Neurog3 long coding isoform: 1,540 bp

ATGCAGCTCAGAAATCCCTCTGGGTCTCATCACTGCAGCAGTGGTCGAGTACCTCCTCGGAGCTTTTCTACGACTTCCAGACGCAATTTACTCCAGGCGAGGGCGC
CTGCAGTTTAGCAGAACTTCAGAGGGAGCAGAGAGGCTCAGCTATCCACTGCTGCTTGACACTGACCCTATCCACTGCTGCTTGTCACTGACTGACCTGCTGCTCT
CTATTCTTTTGAGTCGGGAGAACTAGGATGGCGCCTCATCCCTTGGATGCGCTCACCATCCAAGTGTCCCCAGAGACACAACAACCTTTTCCCGGAGCCTCGGACC
ACGAAGTGCTCAGTTCCAATTCCACCCCACCTAGCCCCACTCTCATACCTAG**G**GACTGCTCCGAAGCAGAAGTGGGTGACTGCCGAGGGACCTCGAGGAAGCTCC
GCGCCCGACGCGGAGGGCGCAACAGGCCCAAGAGCGAGTTGGCACTCAGCAAACAGCGAAGAAGCCGGCGCAAGAAGGCCAATGATCGGGAGCGCAATCGCA
TGCACAACCTCAACTCGGCGCTGGATGCGCTGCGCGGTGTCCTGCCCACCTTCCCGGATGACGCCAAACTTACAAAGATCGAGACCCTGCGCTTCGCCCACAACTA
CATCTGGGCACTGACTCAGACGCTGCGCATAGCGGACCACAGCTTCTATGGCCCGGAGCCCCCTGTGCCCTGTGGAGAGCTGGGGAGCCCCGGAGGTGGCTCCA
ACGGGGACTGGGGCTCTATCTACTCCCCAGTCTCCCAAGCGGGTAACCTGAGCCCCACGGCCTCATTGGAGGAATTCCCTGGCCTGCAGGTGCCCAGCTCCCCATC
CTATCTGCTCCCGGGAGCACTGGTGTTCTCAGACTTCTTGTGAAGAGACCTGTCTGGCTCTGGGTGGTGGGTGCTAGTGGAAAGGGAGGGGACCAGAGCCGTCT
GGAGTGGGAGGTAGTGGAGGCTCTCAAGCATCTCGCCTCTTCTGGCTTTCACTACTTGGATCCCTAGCCCTCTCACAGGGCTTAACTAGGCTTCTCATCGGTACCCT
TGCTGCTGCGCACAGCAGACATTGGGGGCTGCTCTTCTCTTAACTCTCCTCGGTGCAGCCACATCAAACTCTCGCTCCAAGCATTTGAGAATGGTAGCACTACCTA
GTTGGAGACTCCCATACTTCCTGGTGAGTCTGCCCTCATTCAAATCTGCCGGCCTCCGACCATCCATCACTTTTTCCAGGGTGACCTAATCCAGTGTTGCGTCTTACC
TCACTGGCTCCTCCATCCAGCTCTTGGCCCATAGATGATGTTCGTCGTCTTTACTGCCCGCTACATGCAGGGTTTCTGAGCTTCTCCATTCTGCCTTAGTCCACGAAG
GTGATCTGCCTTCTTCTGCACTTTTCAAGTCGTTACCCTTCCCCCAAGGGAGACCAGGCTGTGAACCGGAAAGCCCTAGCCTATGGCTAGAGCATCTCTCCAACTTG
TCTCCCGTGTCTAAAGTGTGAGTTGCAGGGACGGTTCCTGAAGCACTGTTTGTCTCCCT

Neurog3 short non-coding isoform: 1,408 bp

ATGCAGCTCAGAAATCCCTCTGGGTCTCATCACTGCAGCAGTGGTCGAGTACCTCCTCGGAGCTTTTCTACGACTTCCAGACGCAATTTACTCCAGGCGAGGGCGC
CTGCAGTTTAGCAGAACTTCAGAGGGAGCAGAGAGGCTCAGCTATCCACTGCTGCTTGACACTGACCCTATCCACTGCTGCTTGTCACTGACTGACCTGCTGCTCT
CTATTCTTTTGAGTCGGGAGAACTAGGGACTGCTCCGAAGCAGAAGTGGGTGACTGCCGAGGGACCTCGAGGAAGCTCCGCGCCCGACGCGGAGGGCGCAACA
GGCCCAAGAGCGAGTTGGCACTCAGCAAACAGCGAAGAAGCCGGCGCAAGAAGGCCAATGATCGGGAGCGCAATCGCATGCACAACCTCAACTCGGCGCTGGA
TGCGCTGCGCGGTGTCCTGCCCACCTTCCCGGATGACGCCAAACTTACAAAGATCGAGACCCTGCGCTTCGCCCACAACTACATCTGGGCACTGACTCAGACGCTG
CGCATAGCGGACCACAGCTTCTATGGCCCGGAGCCCCCTGTGCCCTGTGGAGAGCTGGGGAGCCCCGGAGGTGGCTCCAACGGGGACTGGGGCTCTATCTACTC
CCCAGTCTCCCAAGCGGGTAACCTGAGCCCCACGGCCTCATTGGAGGAATTCCCTGGCCTGCAGGTGCCCAGCTCCCCATCCTATCTGCTCCCGGGAGCACTGGTG
TTCTCAGACTTCTTGTGAAGAGACCTGTCTGGCTCTGGGTGGTGGGTGCTAGTGGAAAGGGAGGGGACCAGAGCCGTCTGGAGTGGGAGGTAGTGGAGGCTCT
CAAGCATCTCGCCTCTTCTGGCTTTCACTACTTGGATCCCTAGCCCTCTCACAGGGCTTAACTAGGCTTCTCATCGGTACCCTTGCTGCTGCGCACAGCAGACATTG
GGGGCTGCTCTTCTCTTAACTCTCCTCGGTGCAGCCACATCAAACTCTCGCTCCAAGCATTTGAGAATGGTAGCACTACCTAGTTGGAGACTCCCATACTTCCTGGT
GAGTCTGCCCTCATTCAAATCTGCCGGCCTCCGACCATCCATCACTTTTTCCAGGGTGACCTAATCCAGTGTTGCGTCTTACCTCACTGGCTCCTCCATCCAGCTCTT
GGCCCATAGATGATGTTCGTCGTCTTTACTGCCCGCTACATGCAGGGTTTCTGAGCTTCTCCATTCTGCCTTAGTCCACGAAGGTGATCTGCCTTCTTCTGCACTTTT
CAAGTCGTTACCCTTCCCCCAAGGGAGACCAGGCTGTGAACCGGAAAGCCCTAGCCTATGGCTAGAGCATCTCTCCAACTTGTCTCCCGTGTCTAAAGTGTGAGTT
GCAGGGACGGTTCCTGAAGCACTGTTTGTCTCCCT

Supplementary Figure S7. Alternative splicing isoforms of *Neurog3* mRNA. Sequences of detected *Neurog3* splicing isoforms; long coding and short non-coding isoforms. *Neurog3* exons 1, 2 and 3 are in purple, black and green fonts, respectively. Alternative splice acceptor site in exon 2 is underlined and the beginning nucleotide of alternative exon 2 is in bold red font. RT-PCR primers N1, N2, N3 and N4 are highlighted in yellow, green, blue and grey respectively.

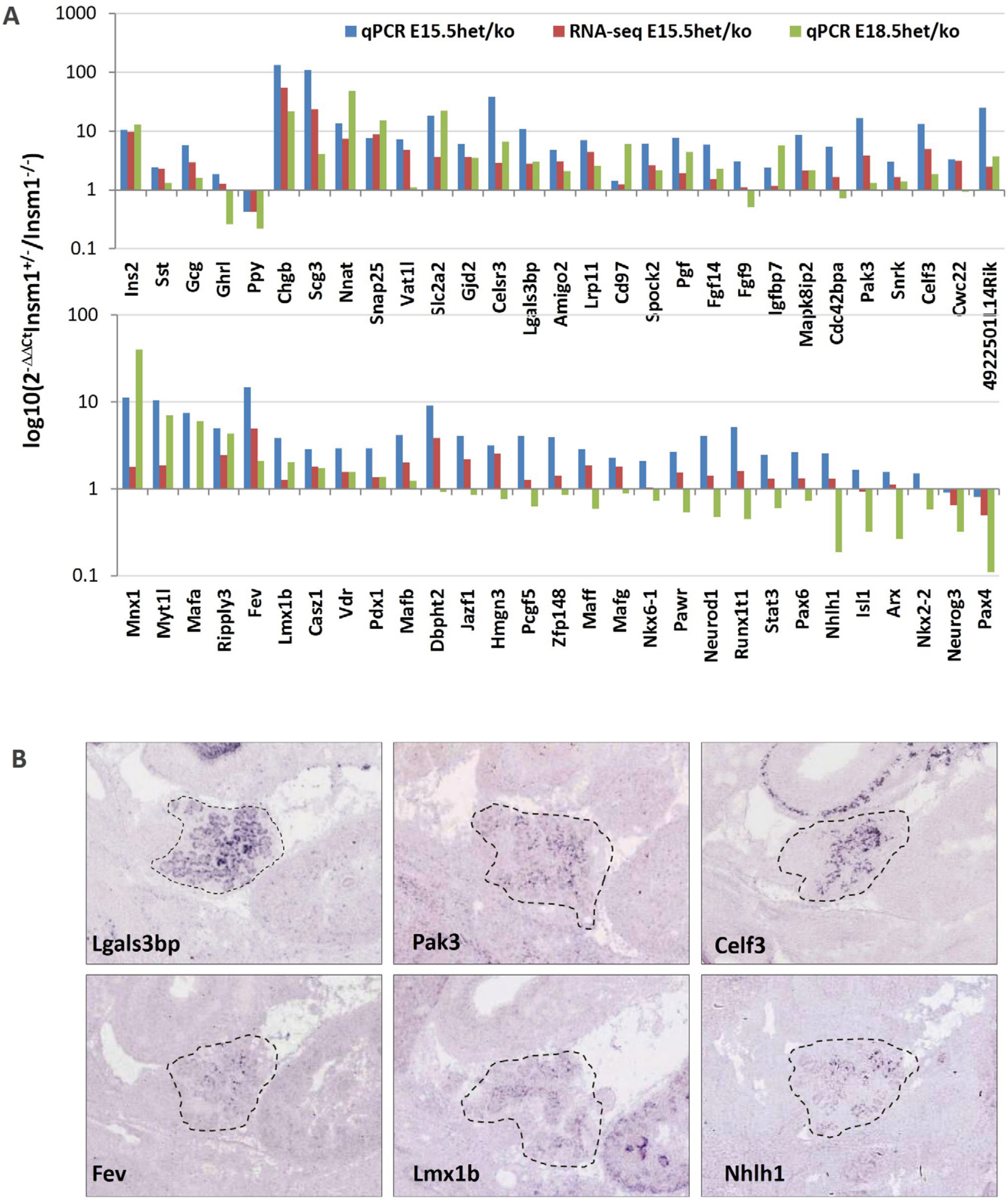

Supplementary Figure S8. Validation of expression of genes upregulated in *Insm1*$^{+/-}$ endocrine cells. (A) Differential gene expression in *Insm1*$^{+/-}$ and *Insm1*$^{-/-}$ endocrine cells was validated by qRT-PCR. Relative gene expression ($2^{-\Delta\Delta Ct}$) was measured for three independent samples of non-amplified RNA at E15.5 and E18.5. Adjusted fold change values obtained by RNA-seq at E15.5 are plotted for comparison. (B) Expression of selected differentially expressed genes is established in developing pancreatic endocrine tissue at E14.5 by in situ RNA hybridization. Pancreas images are obtained from http://www.eurexpress.org.

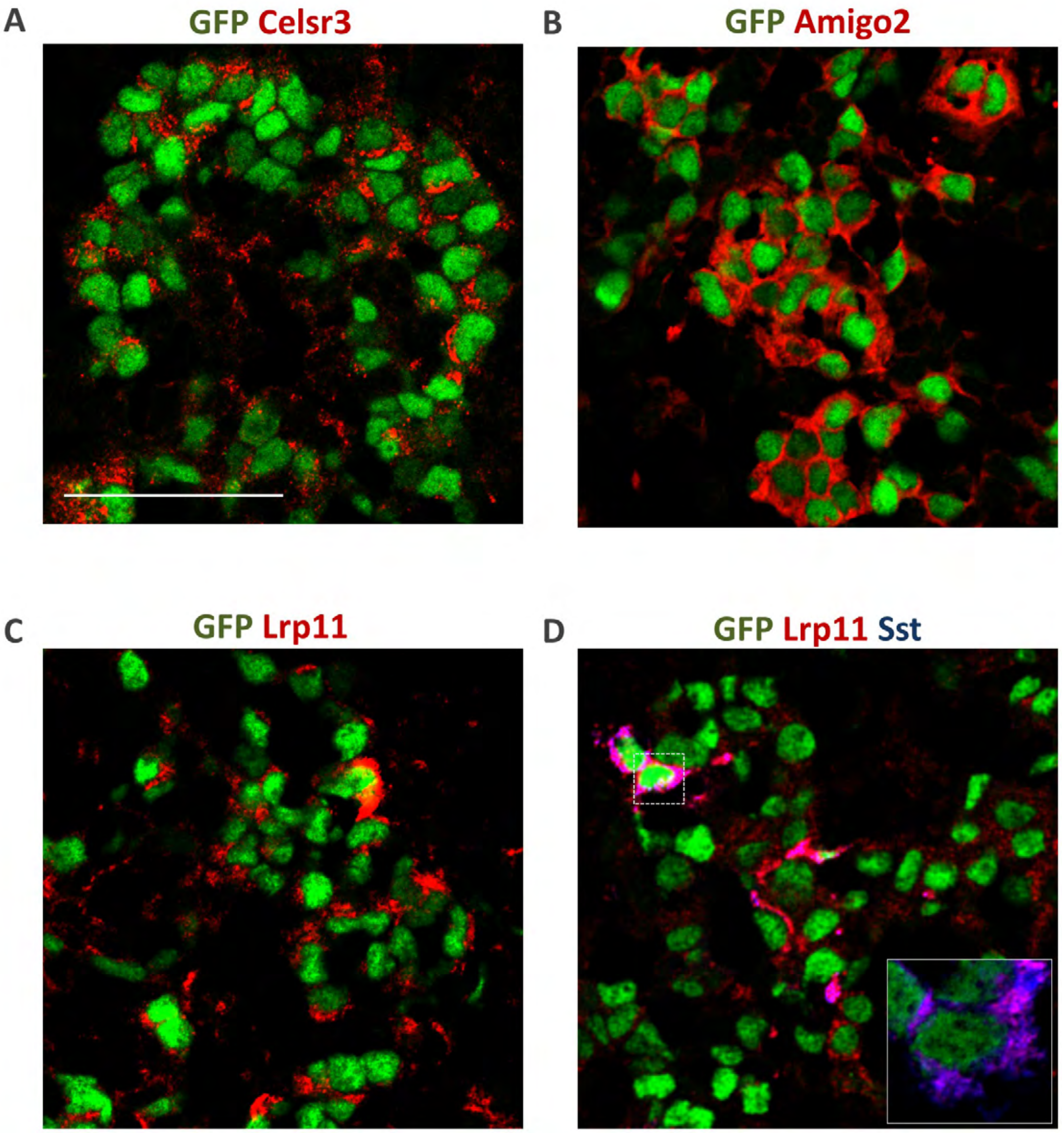

Supplementary Figure S9. Potential cell surface markers for early endocrine cells. *Amigo2 (*adhesion molecule with Ig-like domain 2), *Celsr3* (cadherin, EGF LAG seven-pass G-type receptor 3 (Flamingo homolog, Drosophila)) and *Lrp11* (low density lipoprotein receptor-related protein 11) were identified as genes up-regulated in *Insm1*$^{+/-}$ cells. Immunofluorescence staining with anti-CELSR3 (A), anti-AMIGO2 (B), and anti-LRP11 antibodies (C-D) demonstrates enriched co-staining of GFP-positive endocrine cells in the pancreas of *Insm1*$^{+/-}$ animal at E15.5 (A-C) and E18.5 (D). LRP11 is expressed at higher levels in somatostatin-positive cells (D). Scale bars 50mm.

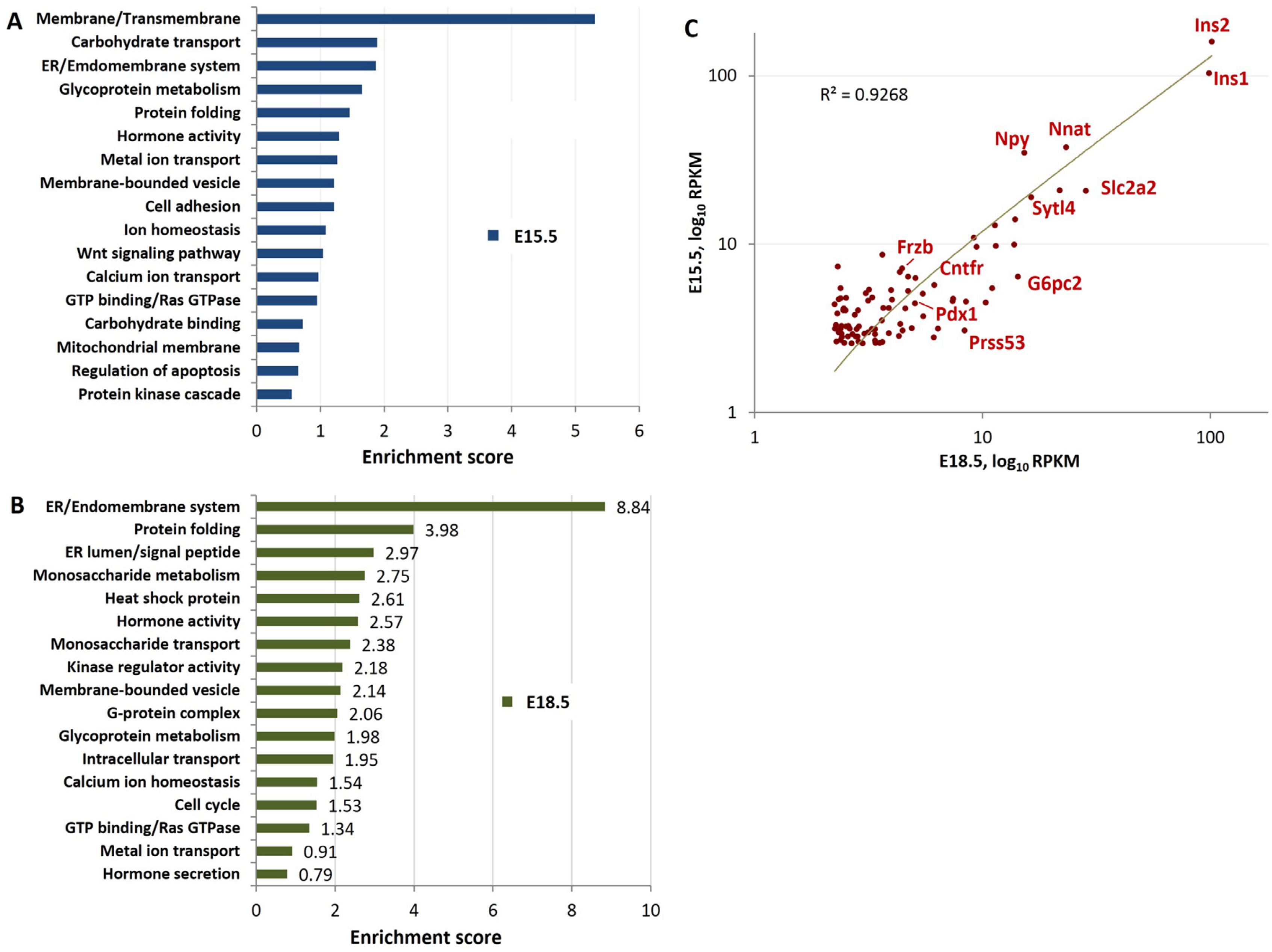

Supplementary Figure S10. RNA-seq analysis of Insm1/Pdx1-HI and Insm1/Pdx1-LO populations at E15.5 and E18.5. (A) Functional groupings of genes up-regulated in *Insm1/Pdx1-HI* versus *Insm1/Pdx1-LO* endocrine populations at E15.5 (A) and E18.5 (B). Enrichment scores for functional gene ontology groups represent the geometric mean (in -log scale) of member's p-values in the corresponding annotation cluster. (C) Correlation plot of RPKM values (log_{10}) for 93 genes commonly up-regulated in *Insm1/Pdx1-HI* at E15.5 and E18.5.

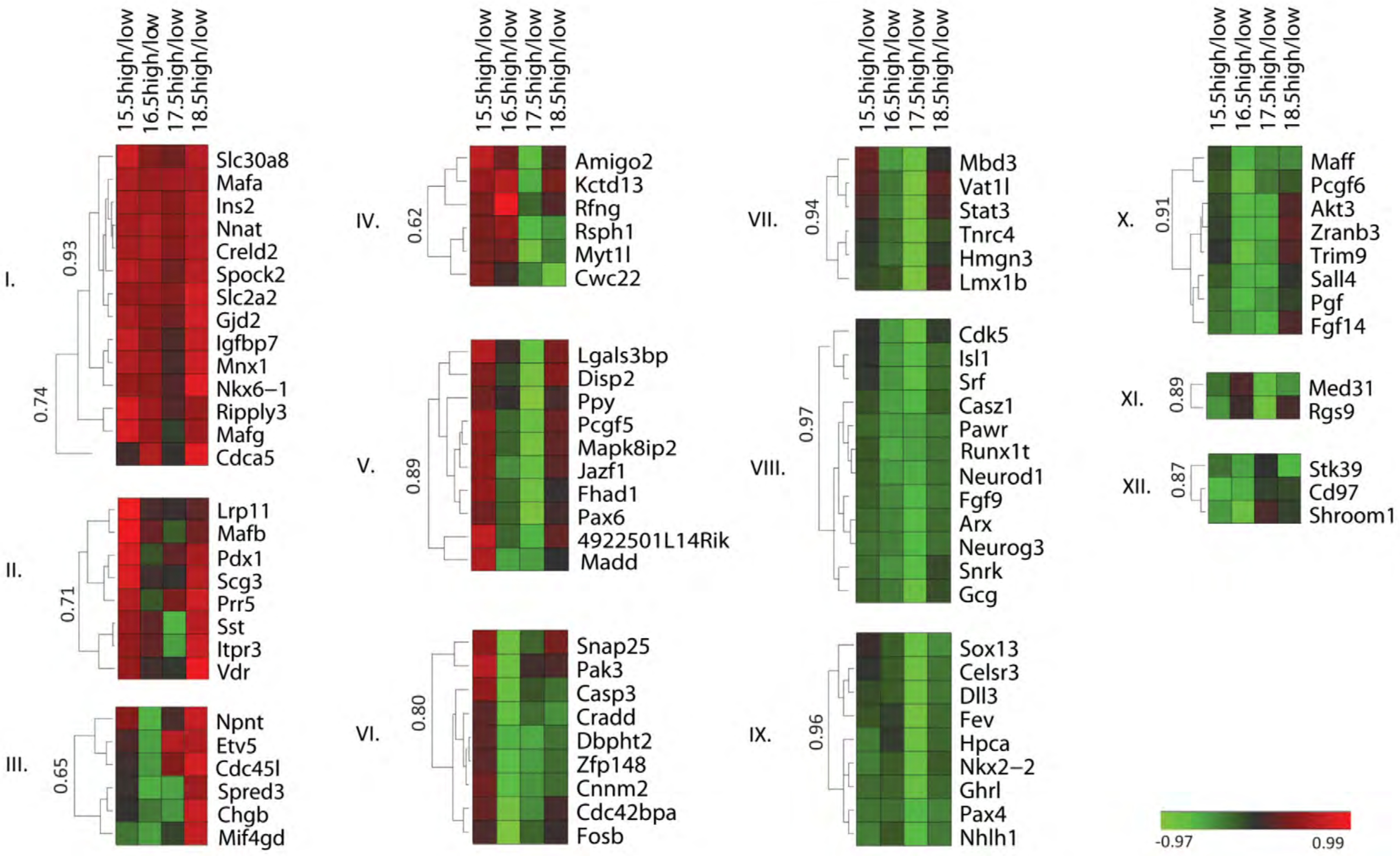

Supplementary Figure S11. Genes differentially expressed in Insm1/Pdx1-HI and Insm1/Pdx1-LO populations. Hierarchical clustering analysis of genes differentially expressed in *Insm1/Pdx1-HI* versus *Insm1/Pdx1-LO* populations based on relative expression (log10) obtained by RT-qPCR. Each row represents the relative levels of expression for a single gene, centered at the geometric mean across all samples. Columns show expression levels for a single sample. Correlation coefficients within clusters are shown on the left of the cluster trees.

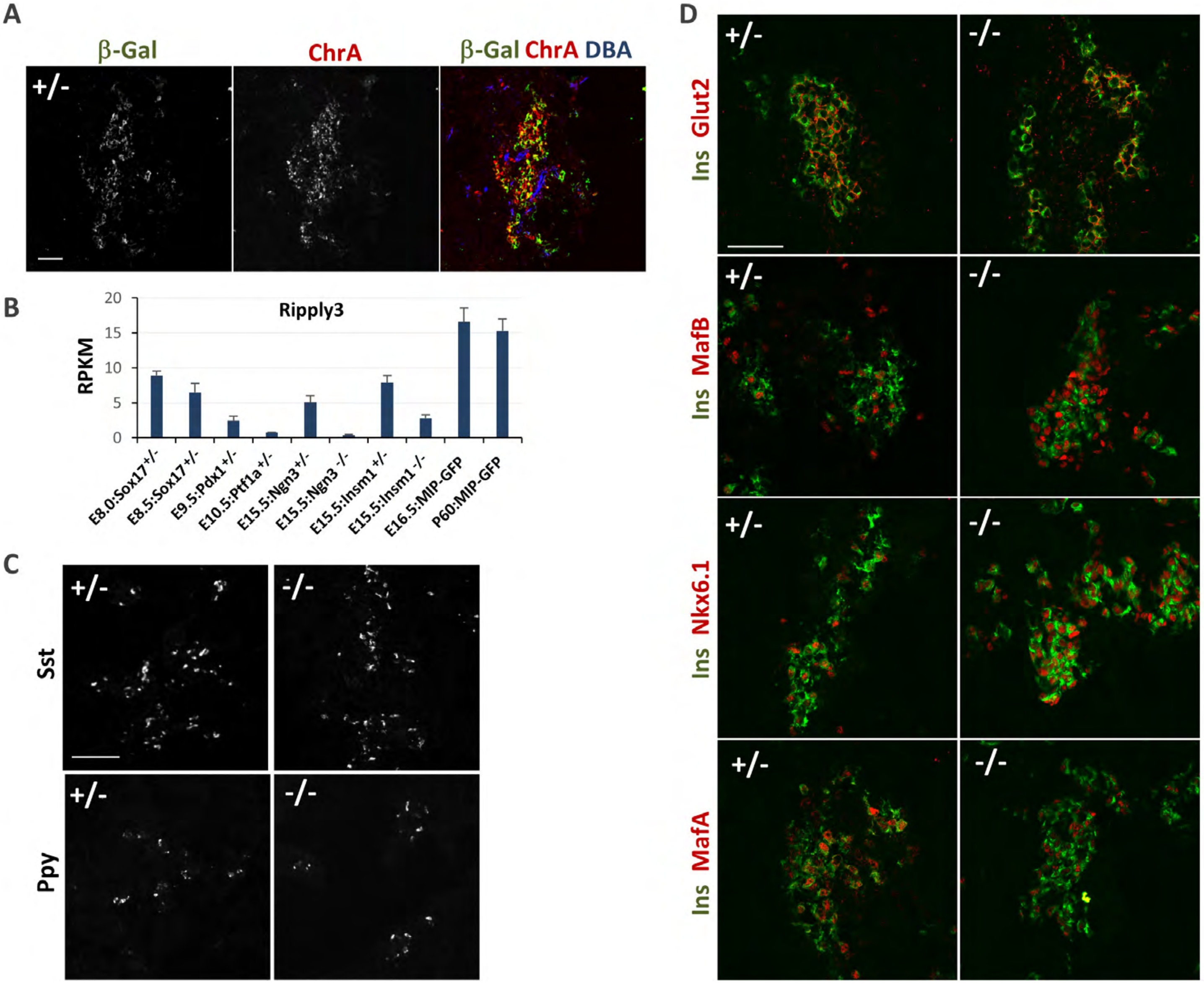

Supplementary Figure S12. Analysis of *Ripply3* in endocrine cells. (A) *Ripply3* is expressed in pancreatic endocrine cells at E18.5. Immunofluorescence staining of pancreata from *Ripply3*$^{\beta\text{-}Gal}$ heterozygous mice with anti-β-galactosidase and antibodies against pan-endocrine chromogranin A. Ductal cells are stained with DBA. (B) Expression of *Ripply3* mRNA in FACS-purified pancreatic developmental intermediate populations as identified by RNA-seq (M.A. Magnuson, unpublished). The populations are as follows, **E8.0 Sox17**$^{+/-}$: Sox17-positive early endoderm (*Sox17*$^{GFP/+}$); **E9.5 Pdx1**$^{+/-}$: Pdx1-positive pancreatic endoderm (*Pdx1*$^{CFP+/-}$); **E10.5 Ptf1a**$^{+/-}$: Pdx1-positive pancreatic multipotent progenitor cells (MPCs) (*Ptf1a*$^{YFP/+}$); **E15.5 Ngn3**$^{+/-}$: *Neurog3*-positive endocrine progenitor cells (*Neurog3*$^{GFP/+}$); **E15.5 Ngn3**$^{-/-}$: *Neurog3*-negative cells (*Neurog3*$^{GFP/GFP}$); **E15.5 *Insm1***$^{+/-}$: *Insm1*-positive pre-endocrine cells (*Insm1* $^{GFP/+}$) ; **E15.5 Insm1**$^{-/-}$: *Insm1*-negative cells (*Insm1*$^{GFP/GFP}$); **E16.5 MIP-GFP**: nascent β-cells; **P60 MIP-GFP**: mature β-cells (*Tg(Ins1-EGFP)*^{1Hara}). RPKM, reads per kilobase per million reads. (C) Immunofluorescence staining for somatostatin (Sst) and pancreatic polypeptide (Ppy) for representative sections of *Ripply3*$^{\beta\text{-}Gal}$ heterozygous and knout mice. (D) Immunofluorescence staining for insulin(Ins) β-cell maturation markers Glut2, MafB, Nkx6.1 and MafA in *Ripply3*$^{\beta\text{-}Gal}$ heterozygous and knockout pancreata at E18.5. Scale bars = 50 mm.

Download Table S1

Supplementary Table S2. Sequences of promoter regions of *Insm1*-regulated and control genes.

Promoter	Sequence*	Predicted Insm1 binding sites location (strand, JASPAR score)
Rest	GATTAGGTAAGTTTCCCCCGAGGCGCGCCCCCAGGCAGGCCAGGAGTCCACTTCGAGCCTCGGGACC<br>GAGGGGCGGGGCGCGCTCGGCGGCCACGCGCGCGGGCGGGGAAGGGGGCGTGTCGGCGGGCGCGC<br>GCGGACGGCGAGGGGGCGTGTCCGGCGGGCGGGCGGGCGGCGACGGCGCGGGCCGGGTGCGCGGC<br>GCAGCGTCCTGTGCTGGAATGTGC	-174/-162 (antisense, 15.448)<br>-94 /-82 (sense, 12.103)<br>-82 /-70 (sense, 9.715)<br>-61/-49 (sense, 10.669)
Cdkn1c	CGCCGAGGGGCTGTGCCCAGCTCCATGGTCCGAGGGGGCGCGCGCGTCGCGGCTGCGCGAGCCCCCG<br>CCTGCAGACAAAGGAGCCCGCGGGGGGCGGGGGGGGGGATGGGGCGAGGGGGAGGGGCGTCGCGG<br>TGTCACGTTACCGCCCGCAGAGCTCTTTAACTCGAGCCCCGCCCCTTGTCCCGCGCAGCCCGCTCGCC<br>GTGCGGCCGCCAATCAGCGCCCGCCCGTCACCCGCCCCGCCCGGCCCCGGGGGTGTGTGCGCGCAGC<br>CAATGAGCGTGGCGCGGGGGCAGGGCCTCTGTGGGCGGGGCGGGCTGCACCCTCCAATCGCCGCGGT<br>GTTGTTGAAACTGAAAATACTACATTATGCTAATCGTGAGGAGGCCCGCGCGCGCGGGGGT	-357/-345 (sense, 8.506)<br>-320/-308 (antisense, 8.264)<br>-276/-264 (sense, 8.641)
Cdkn1b	GAGCGGTCAGTCCTGGCTTCTTTTTGAAACCACCTCCTGGCCTGTCCTACATAGCAGAGACTTCTGGG<br>GTCAGGCTAAGCGGCCGATTCCGCACCCCAGCTAAGGCACCCTGCCGGGTGGGGGTGGGTGGGCTGC<br>CCCAGGCCCTGAGCCAAGGTTTCCTGAATCTCTCGCATACCCTCCTCCATTCCCACCTCGGCGAATGC<br>AGGTGGGCTGAACTAGCCACCGAAGCTCCTAATAAGCCGCCAGCGACCTAGGAGCCCACTGGTCCAG<br>GCCCTAGGTTTCGCGGGCAAAGACCTGGAGGTCGCAGTCGGGAGCGGTGACGCGCCTGCCAGCGCA<br>GCTCTGCAGCGCCGGGACCTAGATCCCCGGGTCCCTGCCTGGCCGCCGCGCTGGCCCCTCCCCAGCTC<br>TCCGGCCGTTTGGCTAGTTTGTTTGTCTTATTTTTAATTTCTCCGGGGCCAGCCAGAGCAGGTTTGTTG<br>GCAGTCGTACACCTCCGAGTAGTCACGCGACCAGCCAATGTCCTGGCGGCGCTACGGGGAGGCGGCG<br>CGCTCGGGAGCGCGGAGAGGCGGCGGCGCCGCGCCCGGGGCCACCTTAAGAGCGCGCTCGCCAGCC<br>TGGGCGGAGCGGCTCCCGGCGCCGAGACCAATGGAGCTCCTCCTCTGTTTAAATAGACTTGC<br>AGTGTCAATC	-638/-626 (antisense, 8.564)<br>-608/-596 (sense, 8.515)<br>-537/-525 (antisense, 9.051)
Ripply3	GCACTGGAGAACAGGTTATTGGGCAAAGGGGTACCCGGGGTCCTGACTGGTGACCCCTAACAGGAA<br>GCACCCCTCCCCCATCTTAAGGCTTTCGTCGGTGCGGAGGTTGCGCCTAAACCCTCGCCTGGCTTCGT<br>AAACCCAGCTGTCACCCACTCGTGGAGCTGTCCTGACCCTCGGTGTAGCGAGGACTCTGTGCCCCAC<br>ACTCACCACCTCTCACAGTGCTGAAAGGGAAACTCTGGGAGAACTGGGGTGCGGGGTGGGGCTTCAT<br>GCTTTTAGGGATCAGAGCCCCGAGGGCAGCGGCCCCAGGAGTTGCTCAGCAGTGGGGAAAATTGTTC<br>CCCGGGACGCGCAGCCGCCACCCGTCATCTCGGAGGCCCTGTAGGCCGTCTGTCCCGCCCTGCGCTTC<br>CCGCTCCGCCAGTGACCCTCCTGGTTTTCTTTTGTCCTTTCTCCACTCTGGACACACACCCCTCCCCGC<br>CGTCCCGACCCTGCTCTCTTCCTTGAGCCTTTCTGGTCTCCACCCTCTGCTTCCTCCGTCCCTTCCCGA<br>GGTCCCTTGCCCTCCCCCTCCCCACGGGGCGCAGTTTCCCCGAGTCCCCTCCCCGCCCCGCCCCAGTT<br>TTTAAACCTGGCGCCGAGTGAGCGGGT	-526/-564 (sense, 13.543)<br>-74/-62 (antisence, 7.515)
Insm1	CCTCGAGTCCCGGCCCCTTTTTTGCCGCTCCGTCGCCCTCCCGCCCTCGTCGGCGGAGGAGCTGCGGA<br>CGCTCTGATTGGCTCTGGGGGAAGCGGGAGGCGAGAACAATGGCCCCCTCCCCCCGTTAAAAGGGAG<br>CGGCGCCCGGGCCCGGGGACAGGGACGCGCGTGCGGAGCGCAGAGCCGGGCCAGCAGCCGCGGGCA<br>CTGCGGGAGTCCCGAAGCCGCCGCGTGCAGGCG	-143/-131 (sense, 7.741)
Pck1	CTGCAGTTTGCATCAGCAACAGGCAGGGTCAAAGTTTAGTCAATCAAATGTTGTGTAAGGACTCACT<br>ATGGCTGATAGAGGGGCCTGAGGCCTCCCAACATTCATTAACAACCACAAGTTCAATCATTATCTCCC<br>TGGAGTTTATTGTGTTAAGTCAGTTCCAAACCGTGCTGACCATGGCTATGATCCAAAGGCCTGCCCCT<br>TACGTCAGAGGCGAGCCTCCGGGTCCAGCTGAGGGGCAGGGCTGTCCTCCCTTCTATATAGTATTTAA<br>AGCAAGGAG	

* Sequences of promoters of the genes potentially regulated by Insm1 were analyzed by JASPAR search tool (Mathelier et al., 2014) for the presence of potential Insm1 binding sites. Predicted Insm1 binding sites on the sense DNA strand are highlighted in red and on the antisense strand in green. First exon sequences are highlighted in blue. The sequences of primers used to amplify precipitated chromatin are underlined.

Reference

Mathelier, A., Zhao, X., Zhang, A. W., Parcy, F., Worsley-Hunt, R., Arenillas, D. J., Buchman, S., Chen, C. Y., Chou, A., Ienasescu, H. et al. (2014). JASPAR 2014: an extensively expanded and updated open-access database of transcription factor binding profiles. *Nucleic Acids Res* **42**, D142-147.

Download Table S3

Download Table S4

Download Table S5
